# JARID1D-dependent androgen receptor and JunD signaling activation of osteoclast differentiation inhibits prostate cancer bone metastasis through demethylating H3K4

**DOI:** 10.7150/thno.104135

**Published:** 2025-01-01

**Authors:** Yaohua Hu, Zhite Zhao, Qinghua Xie, Hui Li, Chenyang Zhang, Xinglin He, Yifan Ma, Caiqin Zhang, Qinlong Li, Changhong Shi

**Affiliations:** 1 Division of Cancer Biology, Laboratory Animal Center, Fourth Military Medical University, Xi'an, Shaanxi 710032, China.; 2 State Key Laboratory of Holistic Integrative Management of Gastrointestinal Cancers, Xi'an, Shaanxi 710032, China.; 3 Department of Urology, Xijing Hospital, Fourth Military Medical University, Xi'an, Shaanxi 710032, China.; 4 Gansu University of Traditional Chinese Medicine, Lanzhou 730030, China.; 5 Department of Pathology, School of Basic Medicine and Xijing Hospital, State Key Laboratory of Cancer Biology, The Fourth Military Medical University, Xi'an, China.

**Keywords:** JARID1D, demethylase, bone metastasis, prostate cancer, osteoclast differentiation

## Abstract

**Rationale:** Bone metastasis and skeletal-related complications are primary causes of mortality in advanced-stage prostate cancer (PCa). Epigenetic regulation, particularly histone modification, plays a key role in this process; however, the underlying mechanisms remain elusive.

**Methods and Results:** In mouse models, JARID1D was an important mediator of both visceral and bone metastases. Chromatin immunoprecipitation (ChIP) and immunofluorescence (IF) techniques showed that the H3K4me3 demethylation activity of JARID1D is a key factor in the dynamic regulation of androgen receptor (AR) expression. Further analysis using western blotting and bone culture systems indicated that knocking down JARID1D enhanced the expression of monoamine oxidase A (MAOA) through the AR signaling pathway, leading to increased secretion of the nuclear factor kappa B (NF-κB) ligand receptor activator (RANKL) by PCa cells. This in turn promotes osteoclast differentiation and facilitates bone metastasis. In addition, single-cell sequencing results indicated that a reduction in JARID1D levels directly affected osteoclasts, stimulated JunD transcription, and accelerated PCa bone metastasis progression. Finally, both *in vivo* and *in vitro* experiments confirmed that the JARID1D agonist JIB-04 effectively blocked these molecular pathways, thereby delaying the onset of bone metastasis in PCa.

**Conclusions:** These insights provide a theoretical foundation for targeting JARID1D and related molecules in the treatment of PCa bone metastasis.

## Introduction

Prostate cancer (PCa) is the second most diagnosed cancer in men and the fifth leading cause of cancer-related mortality, primarily because of its propensity for incurable bone metastasis 1. Bone metastases often exhibit abnormal lytic and osteoblastic characteristics. Although PCa bone metastases are typically characterized as osteoblastic through radiological assessment, patients with high-risk PCa with bone metastases often also exhibit osteolytic characteristics, as evidenced by histological and clinical sample studies 2-4. The interplay between osteoclasts and osteoblasts during bone turnover and remodeling, driven by osteolytic activity, plays a crucial role in the progression of osteoblastic PCa bone metastases 5-7. Notably, the existing treatment options do not significantly improve the prognosis of patients with bone metastatic PCa. Consequently, there is an urgent need to identify factors that regulate bone metastasis in PCa.

Bone metastasis is a multistep process involving complex interactions between tumor cells and the bone microenvironment 8. Epigenetic regulation, particularly alterations in histone modification, plays a pivotal role in this process. In the context of PCa bone metastasis, specific changes in histone modifications have been shown to exert a significant influence on the invasiveness and metastatic potential of tumor cell 9. Concurrently, compounds that target histone modifications have been introduced into clinical trials, including EZH2 10, LSD1^11^, BET 12, HDAC 13, and DNMT 14. Therefore, targeting epigenetic regulatory factors may be a novel therapeutic strategy to combat bone metastasis in PCa.

JARID1D, a male-specific histone demethylase, plays a crucial role in curbing tumor invasion by demethylating the histone marker H3K4me3 15. JARID1D selectively targets the promoters of genes implicated in invasion and metastasis, such as metalloproteinase (MMP)1 and MMP3, thereby diminishing the invasive capacity of tumor cells and impeding metastatic process 16. Furthermore, knockdown of JARID1D induces high expression of key regulatory molecules involved in epithelial-mesenchymal transition (EMT), such as N-cadherin and Slug 16. In metastatic PCa, reduced or absent JARID1D expression is associated with poor prognosis 16. However, the comprehensive role of JARID1D in PCa, particularly in bone metastasis, has not been fully elucidated.

In the present study, we hypothesized that JARID1D modulates the differentiation and functionality of osteoclasts to influence the bone metastasis of PCa by altering the state of histone modifications and affecting the expression of related genes. Single-cell sequencing revealed that JARID1D was involved in cell migration, cell adhesion, and osteoclast differentiation. The gradual loss of the AR during the development of PCa from castration-resistant prostate cancer (CRPC) to neuroendocrine prostate cancer (NEPC) is a pivotal catalyst for bone metastasis. JARID1D modulates AR activity by H3K4me3 demethylation, inhibiting MAOA and decreasing PCa cell secretion of RANKL, which inhibits osteoclast differentiation and bone metastasis. Moreover, JARID1D directly influences osteoclasts by modulating the activity of the osteoclast differentiation-related transcription factor JunD, thereby inhibiting bone metastasis in PCa. The JARID1D agonist effectively inhibits bone metastasis in a mouse model of PCa. These findings provide molecular insights into the role of JARD1D in PCa progression and highlight its potential as a therapeutic target for PCa metastasis.

## Materials and Methods

### Human samples

Human PCa samples were obtained from the Xijing Hospital of the Fourth Military Medical University (Xi'an, China). Written informed consent was obtained from the patients, and all study protocols were conducted in accordance with the guidelines approved by the Xijing Hospital Clinical Research Ethics Committee (No. KY20173028-1). The representative clinical patient case information is presented in table S1.

### Cell lines, antibodies, and expression plasmids

The DU145, 22RV1, PC3, C42, and LNCaP prostate cell lines, mice macrophage RAW264.7, and MC3T3-E1 subclone 14 cells were purchased from the American Type Culture Collection (ATCC; Manassas, VA, USA), which confirms cell lines using short tandem repeat analysis. Cell culture reagents were purchased from Invitrogen/Gibco. Antibodies used were as follows: Anti-JARID1D (Affinity DF2548), anti-AR (Abcam, ab108341) and (santa cruz, sc-7305), anti-MAOA (Novus Biologicals, NBP2-38868), anti-H3K4me3 (Affinity, DF695), anti-JunD (santa cruz, sc-271938), anti-RANKL (Proteintech, 23408-1-AP), Osteocalcin (servicebio, GB115684), OPG (servicebio, GB115706), PGK1 (Proteintech, 17811-1-AP), Substance P (servicebio, GB11412). Normal Rabbit IgG was obtained from Santa Cruz Biotechnology (Santa Cruz, CA, USA; sc-2027). Anti-β-actin (AT0001) antibodies were purchased from EngiBody. Both the JARID1D agonist JIB-04 (HY-13953) and JunD inhibitor T-5224 (HY-12270) were purchased from MCE (USA, NJ, USA). The AR agonist R1881 (M8128) was purchased from AbMole. JARID1D and its catalytic mutant (HE/AA) were cloned into the pFlag-CMV2 vector (Sigma-Aldrich). JARID1D catalytic mutant mJARID1D (HE/AA) in recombinant baculoviral form is enzymatically inactive 15.

### Human PCa cell inflammatory cytokine analysis

Human PCa cell inflammatory cytokines were detected by Wayen Biotechnology Co., Ltd. (Shanghai, China), as previously reported 17. Briefly, supernatant samples were collected from control and knockdown cells. The Human 27 Cytokine Array was used to couple highly specific captured monoclonal antibodies to different fluorescently labeled magnetic beads. The different types of magnetic beads were mixed and resuspended in 96-well microplates. A biotin-labeled high-affinity paired detection antibody was added to amplify the signal and achieve effective cytokine detection.

### Ectopic expression experiments

Through lentiviral transduction, we successfully generated stable cell lines with diminished JARID1D expression, designated 22RV1 Sh-JARID1D and DU145 Sh-JARID1D, to ectopically express JARID1D and its catalytic mutant mJARID1D. We transfected DU145 Sh-JARID1D, 22RV1 Sh-JARID1D, and PC3 PCa cells with the JARID1D or mJARID1D cDNA construct using Lipofectamine 3000 (Thermo Fisher Scientific, L3000008), according to the manufacturer's instructions. After 24-48 h of incubation, the cells were harvested and total RNAs was isolated for further analysis.

### Cell invasion assays

DU145, 22RV1, and PC3 cells (1.0 × 10^5^) were suspended in Roswell Park Memorial Institute (RPMI) 1640 containing 0.1% bovine serum albumin (BSA) and seeded onto Matrigel-coated membranes in each insert. After 48 h, the cells that invaded the Matrigel and moved to the other side of the membrane were fixed.

### Western blotting and immunoprecipitation (IP)

Western blotting and IP were performed as previously described 18. For western blotting, the cells were lysed in lysis buffer and sonicated. Proteins were separated by electrophoresis, transferred onto a membrane, blocked, incubated with antibodies, and visualized using an enhanced chemiluminescence reagent. For IP, the cells were washed twice with phosphate-buffered saline and scraped into IP lysis buffer, according to the manufacturer's instructions. Total cell lysates were precleared by incubation with protein G-linked agarose beads at 4 °C for 2 h. After preclearing, the lysates were incubated with the primary antibody overnight at 4 °C and then with protein G-linked agarose beads for 1 h at 4 °C. The beads were washed thrice with IP buffer, and the protein-antibody complexes were eluted from the agarose and detected.

### Chromatin immunoprecipitation-quantitative polymerase chain reaction (ChIP-qPCR)

ChIP-qPCR was performed as previously described 19. The cells were fixed with 37% formaldehyde, resuspended in ChIP lysis buffer, and sonicated. The chromatin lysates were incubated with antibodies against H3K4me3 or IgG overnight at 4 °C, followed by the addition of protein G-coupled agarose beads. Protein-chromatin complexes were extracted from the agarose beads using an elution buffer. Reversal of cross-linking between proteins and DNA was achieved by incubating the elution buffer with 10 mg/mL RNase A and 5 mM NaCl overnight at 65 °C, followed by incubation with 0.5 mM ethylenediaminetetraacetic acid (EDTA), 1 mM Tris, pH 6.5, and 10 mg/mL proteinase K for 1 h at 45 °C. Purified DNA was quantified in triplicate using SYBR green for quantitative reverse transcription (qRT)-PCR. The primer sequences used for PCR are listed in table S2.

### Biochemical analyses

Human RANKL and mouse IL-2, IL-4, IL-6, TNF, IFN-γ, and IL-1β secretion in cell-culture media were quantified using the species-specific enzyme-linked immunosorbent assay (ELISA) from USCN life science, according to the manufacturer's protocols. Standard immunohistochemistry (IHC) and hematoxylin and eosin (H&E) staining were performed as previously described 20. For Immunofluorescence (IF), cells were incubated with specific JARID1D and AR antibodies after fixation, permeabilization, and blocking. DAPI was used to stain the cell nuclei and the results were obtained using a fluorescence microscope. Tartrate-resistant acid phosphatase (TRAP) staining was performed on osteoclasts derived from RAW264.7 macrophages and bone marrow cells obtained from C57BL/6J mice, as previously described 21. Staining was conducted on day 6 or 9 using a leukocyte acid phosphatase kit (Servicebio, G1050-50T). Multinucleated cells exhibiting TRAP positivity were identified as mature osteoclasts and subsequently analyzed quantitatively. An Alizarin Red staining kit (OriCell, OILR-10001) was used to evaluate the osteogenic differentiation capability of the osteoblasts. Burgundy color represents calcified cells.

### Animal experiments

MAOA wild-type (*MAOA*-WT) and MAOA gene knockout mice (*MAOA*-KO) were purchased from Caygen Biotechnology (Guangzhou, China). Male BALB/c nude mice (5-6 weeks old) were purchased from Gempharmatech Co. Ltd. (Chengdu, China) and bred in a barrier environment at the Laboratory Animal Center of the Fourth Military Medical University (FMMU). All experimental animal protocols were approved by the Institutional Animal Care and Use Committee of the FMMU (Protocol No. 20200417). PCa cells (100 µL, 2 × 10^6^ cells/mL) were injected into the tail veins of nude mice to establish a model of lung metastasis. After 4 weeks, metastasis was observed *in vivo* using small-animal optical imaging (Caliper Lumina II small-animal optical imaging system). When a metastatic signal appeared, lung tissue sections were removed for H&E staining.

For bone metastasis studies, 100 μL of 1 × 10^6^ PCa cells are injected into the left ventricle of anesthetized mice. After 2 weeks, metastatic signals were detected using small-animal optical imaging through radiography (Caliper Lumina II small-animal optical imaging system). When a bone metastasis signal appeared 6-8 weeks, the mice were euthanized, and the bone was removed and fixed for H&E staining, TRAP staining, and IHC analysis.

To determine the therapeutic effect of JIB-04 on metastasis, we injected 100 μL of 1 × 10^6^ DU145-sh JARID1D cells intracardially into mice and 50 μL of 2 × 10^5^ PC3 cells into the left tibial cavity of the mice. Mice were randomly assigned to two treatment groups (JIB-04 vs. control). Daily intraperitoneal injections of JIB-04 (50 mg/kg) were administered for up to 6 weeks, with saline injection in the control group. The radiographs of each mouse were monitored weekly. Images were captured using a Xenogen IVIS50 imaging system, and the bioluminescence signal was measured using Living Image 4.7.3 software.

### Statistical analysis

The data are presented as mean ± standard error (SE), and were analyzed using GraphPad Prism (GraphPad Software, San Diego, USA). Differences between experimental groups were tested using Student's t-test and one- and two-way analysis of variance (ANOVA) (P<0.05).

## Results

### Downregulation of JARID1D is associated with PCa metastasis and poor prognosis

**Figure 1:**
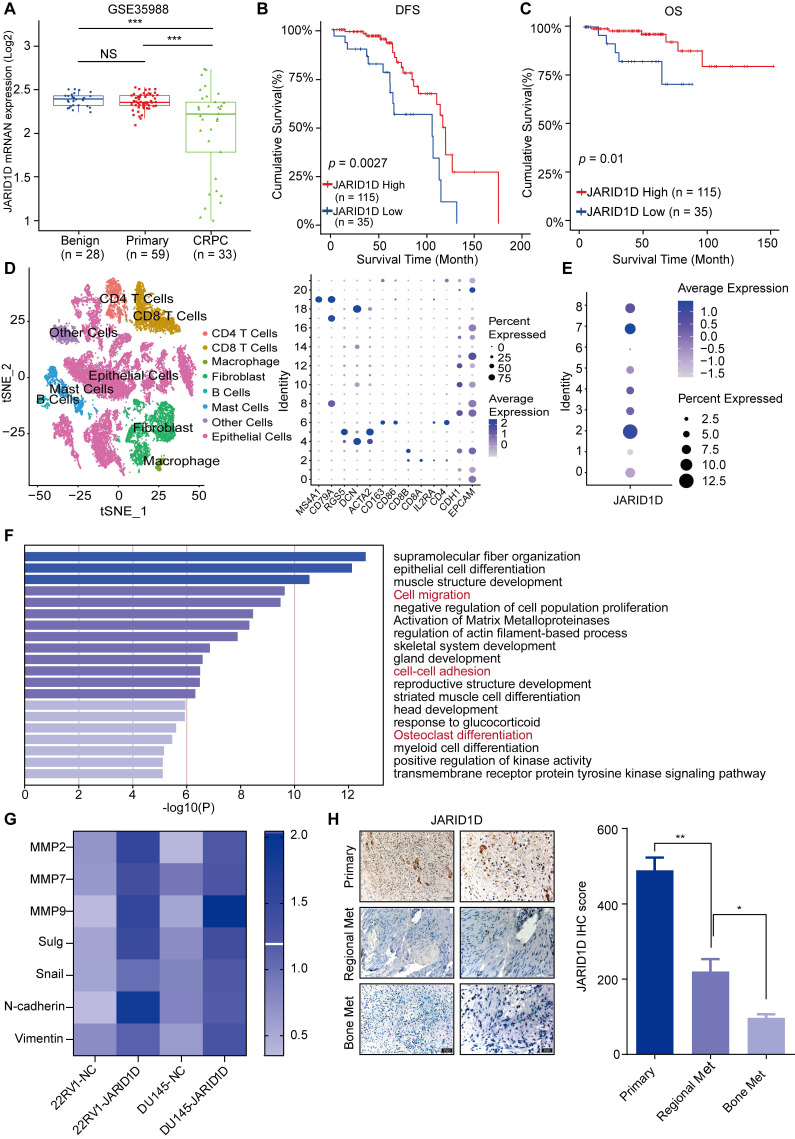
** JARID1D downregulation is associated with PCa metastasis and poor prognosis.** (A) JARID1D mRNA levels in PCa tissues were assessed by analyzing the GSE35988. (B and C) Kaplan-Meier analysis of disease-free survival (DFS) and overall survival (OS) curves for patients with PCa with low JARID1D expression vs. those with high JARID1D expression (log-rank test, n = 150). (D) Cell type annotation of 25782 cells using t-distributed stochastic neighbor embedding (t-SNE) and uniform manifold approximation and T-SNE plots showing average expression of gene markers for all cell clusters. (E) T-SNE plot of JARID1D showing normalized expression levels in the sub-clusters. (F) Gene Ontology (GO) enrichment analysis for JARID1D-high-and JARID1D-low-expression groups. (G) Quantitative reverse transcription-polymerase chain reaction (RT-PCR) analysis of the effect of JARID1D knockdown on the expression of invasiveness-associated genes. (H) Immunohistochemistry (IHC) analysis and quantification of JARID1D expression levels in clinical samples of primary prostate cancer (PCa), local metastatic PCa, and bone metastatic PCa. Scale bars, 50 and 20 μm.

**Figure 2:**
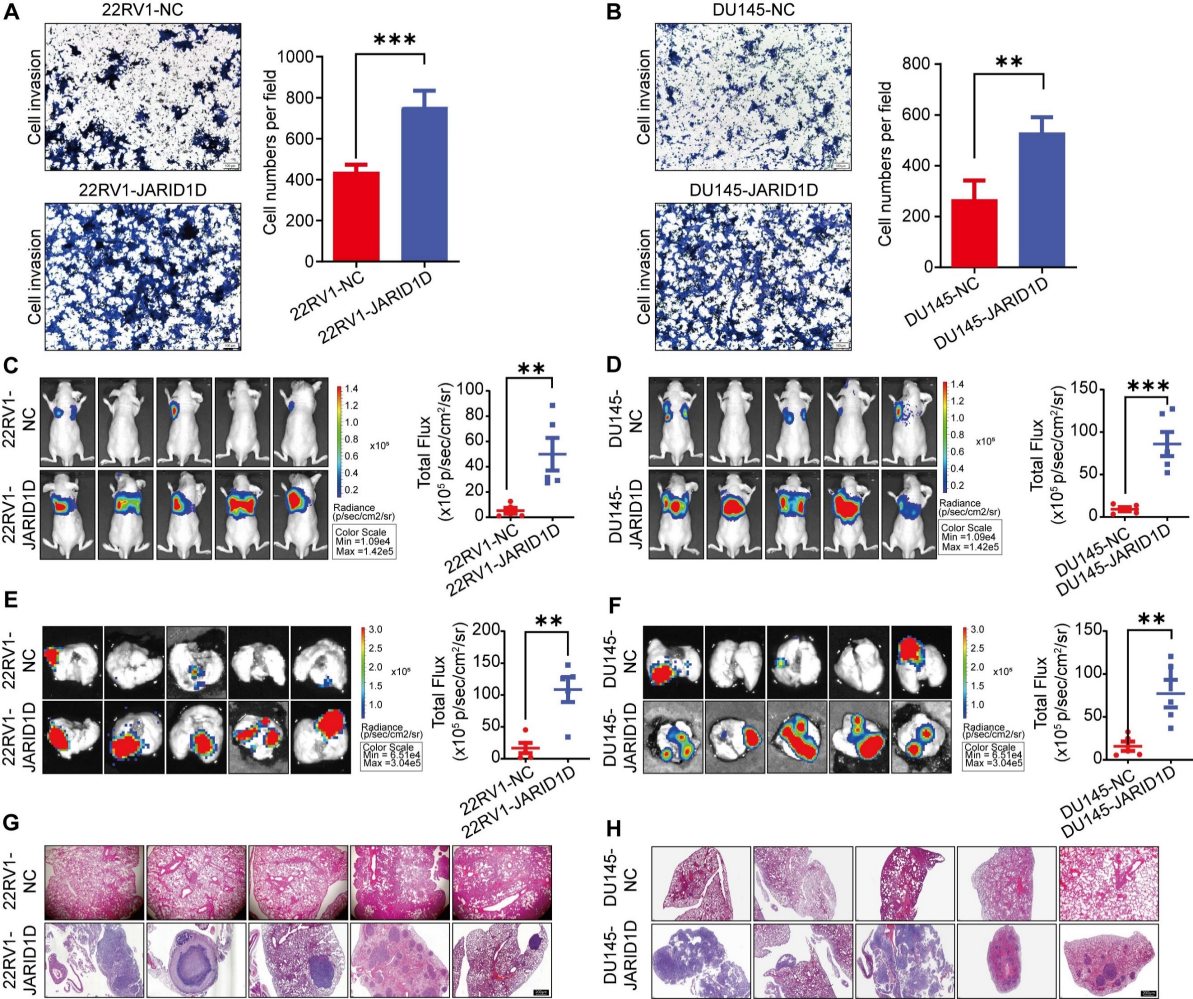
** Downregulation of JARID1D enhanced the invasiveness and metastatic potential of PCa.** (A and B) Transwell and its quantification analyzed changes in invasion ability of 22RV1 and DU145 cells after knockdown of JARID1D compared with NC group. Scale bars, 50 µm; (C and D) Bioluminescence image and its quantification of whole mouse after knockdown of JARID1D in 22RV1 and DU145 cells; (E and F) Bioluminescence image and its quantification of mouse lung tissue after knockdown of JARID1D in 22RV1 and DU145 cells; (G and H) H&E staining results of lung tissue of nude mice after knockdown of JARID1D in 22RV1 and DU145 cells. (*P < 0.05, **P < 0.01, and ***P < 0.001. PCa, prostate cancer; NC, normal control). Scale bars, 200 μm.

To explore the role of JARID1D in PCa progression, our analysis of GEO data revealed stepwise downregulation of JARID1D as the disease became more aggressive (Figure 1A). In addition, patients with low JARID1D expression exhibited a poor prognosis (Figures 1B and C). These findings indicate the clinical significance of JARID1D in PCa progression and suggest that the absence of JARID1D can serve as an indicator of a negative prognosis in PCa. Subsequently, we conducted a comprehensive reanalysis of the single-cell transcriptomic data (GSE137829) for PCa, categorizing 25,782 individual cells into 21 distinct primary cell populations (Figure S1A). These populations were categorized into eight coherent subgroups based on the differential expression profiles of key genes (Figure 2D). Based on the expression profile of JARID1D, the tumor epithelial cells were divided into eight distinct subgroups (Figure 1E, Figure S1B). Functional enrichment analysis revealed that JARID1D was involved in cell migration, cell adhesion, skeletal system development, osteoclast differentiation, and other critical functions (Figure 1F).

To validate the single-cell sequencing results, we initially established a stable cell line with reduced JARID1D expression using 22RV1 and DU145 cells (Figures S1C-E). Subsequent RT-PCR analysis revealed significant upregulation in the expression of genes associated with cell migration and adhesion (Figure 2G). To establish a model of PCa metastasis, 22RV1 cells were injected subcutaneously into nude mice to create tumor-bearing mice*,* and various concentrations of the AR antagonist Enzalutamide (ENZ) solution were administered (Figure S1F). Drug-resistant tumors from the P5 generation were implanted into nude mice and treated with a higher dose of ENZ (25 mg/kg). After 1 month, metastasis to the left femur was observed (Figure S1G). IHC staining indicated a significant decrease in JARID1D expression within metastatic tumors (Figure S1H), which aligns with the results of our bioinformatics analysis (Figure S1I). In clinical specimens, JARID1D was reduced in regional metastasis, and an even more pronounced decrease in bone metastatic prostate tumors than in primary PCa was observed (Figure 1H). Furthermore, we compared the expression levels of JARID1D in primary tumors between patients with and without bone metastasis. Lower JARID1D expression was correlated with an increased likelihood of bone metastasis. (Figure S1J). Collectively, this evidence demonstrates that JARID1D expression is closely linked to the invasive and metastatic capabilities of PCa.

### Downregulation of JARID1D enhanced the invasiveness and metastatic potential of PCa

To elucidate the mechanisms by which JARID1D influences PCa progression, we conducted Transwell migration and invasion assays. The results indicated that, compared to NC cells, the knockdown of JARID1D significantly enhanced the invasiveness and metastatic potential of 22RV1 and DU145 cells (Figures 2A and B). To validate these results *in vivo*, we injected NC and Sh-JARID1D cells into nude mice. After a 4-week observation period, luciferase signals in the Sh-JARID1D groups were significantly higher than those in the NC group (Figures 2C and D), with the metastatic signal predominantly localized to the lungs (Figures 2E and F).

At the end of the experiment, the mice were euthanized and their lungs were dissected. H&E staining revealed that the metastatic foci in the Sh-JARID1D groups were significantly more pronounced than those in the NC group (Figures 2G and H). In summary*,* these *in vivo* findings validated the results of our* in vitro* functional studies, confirming the role of JARD1D as a critical regulator of PCa metastasis.

### JARID1D enzymatic activity is essential for its anti-invasive function

**Figure 3:**
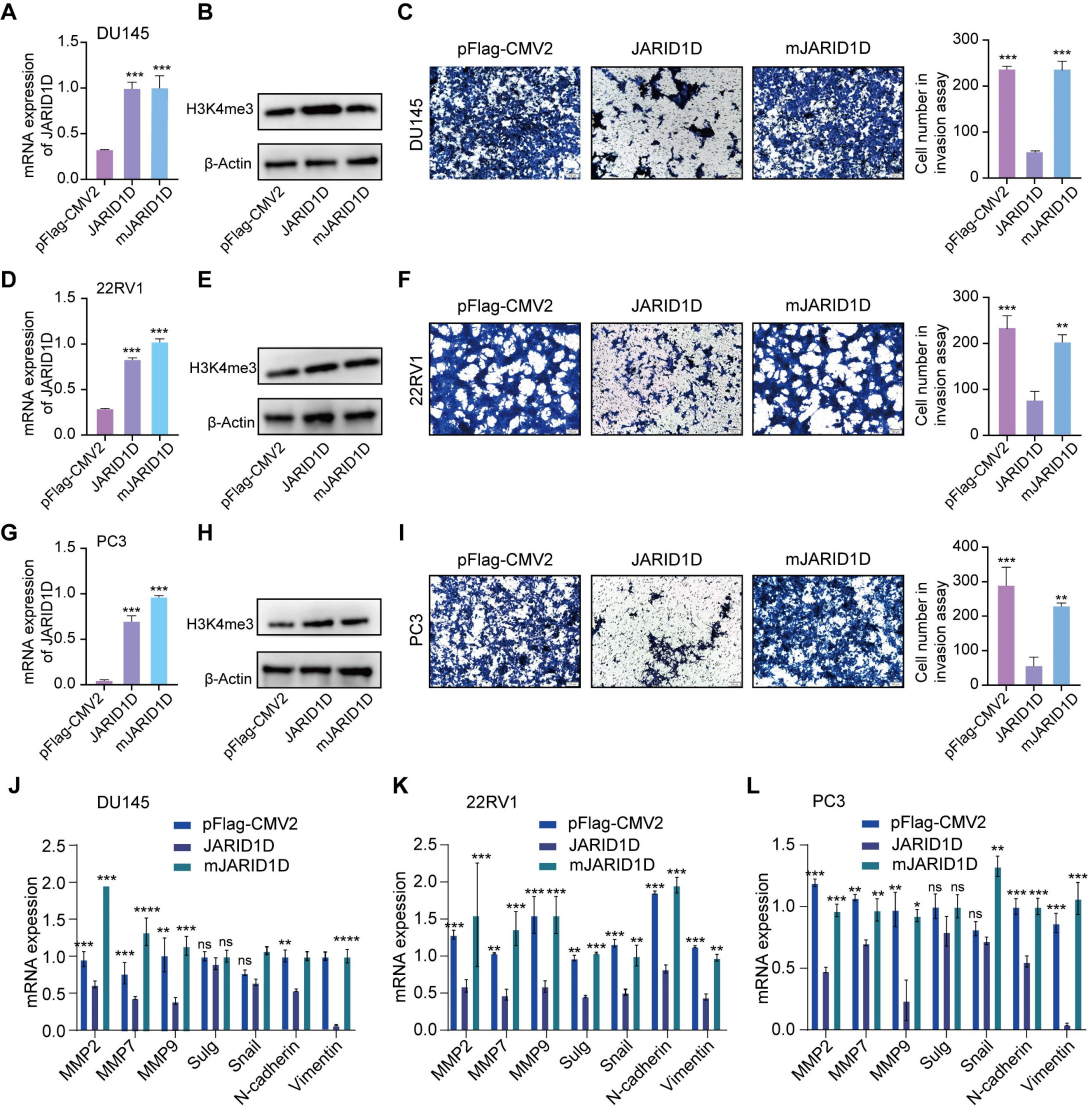
** The enzymatic activity of JARID1D is essential for its anti-invasive function.** (A, D, and G) Ectopic expression of WT JARID1D and its catalytic mutants (mJARID1D) in DU145 Sh-JARID1D, 22RV1 Sh-JARID1D, and PC3 prostate cancer cells. Cells were transfected with control plasmids (pFLAG-CMV2), pFLAG-CMV2-JARID1D, or pFLAG-CMV2-mJARID1D. JARID1D and mJARID1D mRNA levels were measured using quantitative RT-PCR; (B, E, and H) JARID1D protein and H3K4me3 levels were assessed using western blotting; (C, F, and I) Effects of ectopic expression of JARID1D and mJARID1D on the invasiveness of DU145 Sh-JARID1D, 22RV1 Sh-JARID1D. and PC3 cells. Representative images are shown. (J-L) Effects of JARID1D and mJARID1D on invasiveness-associated genes expression in DU145 Sh-JARID1D, 22RV1 Sh-JARID1D, and PC3 cells.

Histone demethylases modulate cellular functions through both enzymatic and non-enzymatic mechanisms. The demethylase activity of UTX, a H3K27 demethylase, is pivotal for the invasive behavior of breast cancer cells 22. In contrast, the development of mouse embryos and expression of T-box family members appears to be independent of the enzymatic functions of UTX 23. To assess whether the enzymatic activity of JARID1D is necessary for cellular invasiveness and to extend the shRNA-mediated JARID1D knockdown experiments, we introduced WT JARID1D and its catalytically inactive mutant mJARID1D into 22RV1 Sh-JARID1D, DU145 Sh-JARID1D, and PC3 cells. As shown in Figures 3A and B, both JARID1D and mJARID1D were overexpressed to a similar extent. This overexpression did not significantly alter the overall levels of H3K4me3 within the cells, which was consistent with the 22RV1 Sh-JARID1D (Figures 3D and E) and PC3 (Figures 3G and H) cell lines.

Transwell assays revealed that JARID1D, but not its mutant form, mJARID1D, significantly decreased the invasive capabilities of DU145 Sh-JARID1D, 22RV1 Sh-JARID1D, and PC3 cells (Figures 3C, F, and I). In agreement with these findings, ectopic expression of JARID1D, but not mJARID1D, led to a marked reduction in the expression of genes associated with invasion and migration in these cells (Figures 3J-L). These findings underscore the necessity of JARID1D and its catalytic activity in invasive PCa cells.

### JARID1D regulates osteoclast differentiation and bone metastasis in PCa

Cytokines secreted by tumor cells, including interleukins (IL) and tumor necrosis factor (TNF), influence not only tumor cells but also osteoblasts, osteoclasts, and other bone cells, thereby promoting bone metastasis 24. Therefore, we investigated whether the expression and enzymatic activity of JARID1D in tumor cells mediates cytokine secretion and contributes to bone metastasis in PCa.

**Figure 4:**
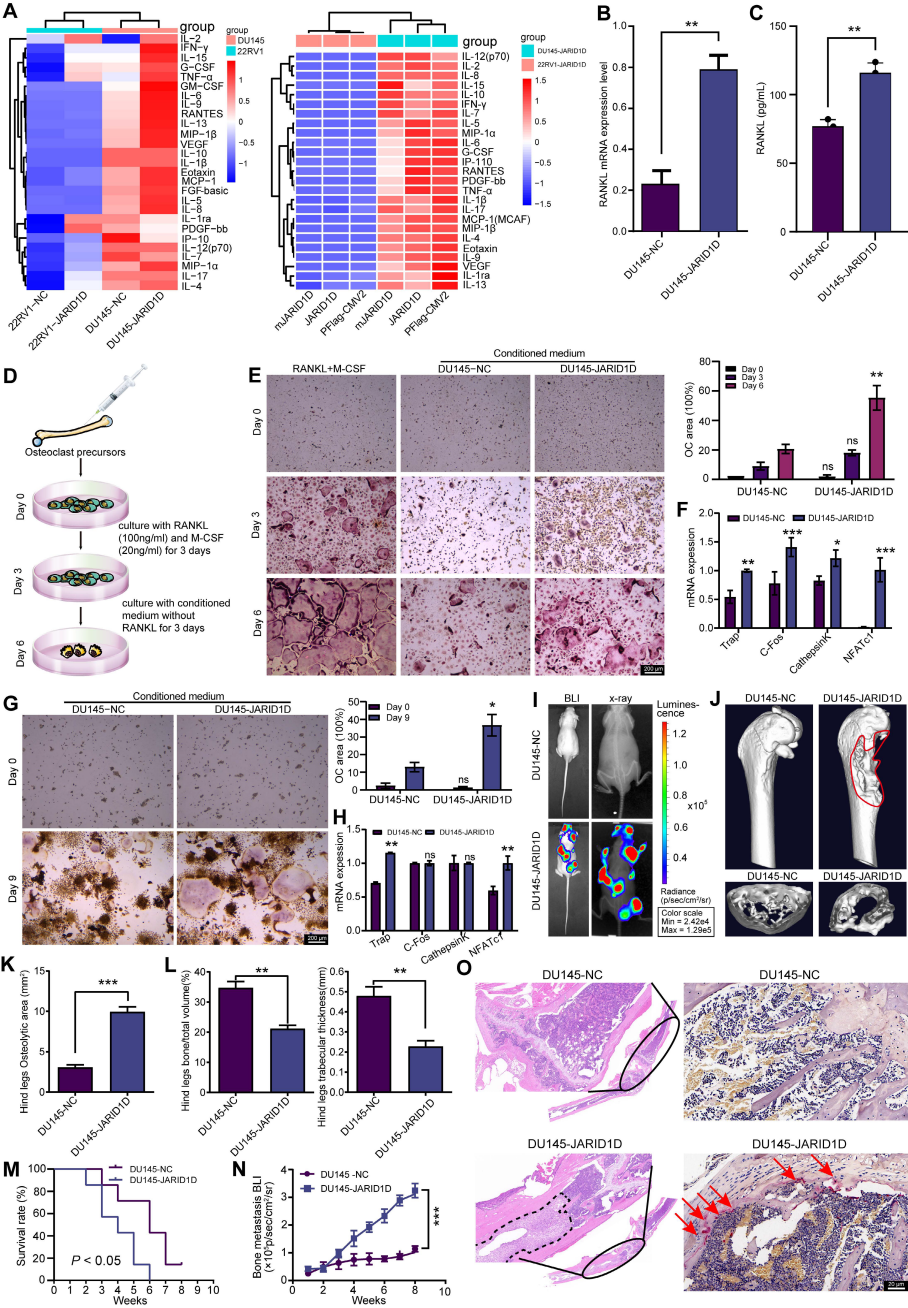
** JARID1D regulates osteoclast differentiation and bone metastasis in PCa.** (A) Cell inflammatory cytokine assay to detect the effects of knocking down JARID1D and its enzymatic activity mutants in 22RV1 and DU145 on the expression of 27 cytokines. (B) RT-PCR detection of RANKL expression in DU145 cells with JARID1D knockdown. (C) ELISA detection of RANKL content in the culture supernatant of DU145 cells after JARID1D knockdown. (D) Schematic diagram of primary osteoclast culture. (E) T tTartrate resistant acid phosphatase (TRAP) staining to assess the differentiation capacity of osteoclasts in different treatment groups and quantification results. (F) RT-PCR analysis of osteoclast differentiation-related gene expression across various treatment groups. (G) Representative TRAP staining images and quantification results of osteoclast differentiation induced by RAW264.7 cell in different treatment groups. (H) RT-PCR analysis of osteoclast differentiation-related gene expression across various treatment groups; (I) Bioluminescence image (left) and radiography image (right) of DU145-NC and DU145 Sh-JARID1D cells intracardially injected into nude mice. (J) Micro-CT image of tibia of hindlimb of representative nude mouse and cross section of tibia of hindlimb of representative nude mouse in DU145-NC and DU145 Sh-JARID1D groups. (K-L) Quantitative map of osteolytic area (K) relative bone volume and trabecular thickness (L) of tibia of hindlimb in (H and I) above (n = 3); (M) Survival analysis comparing the intracardiac injection groups of DU145-NC and DU145 Sh-JARID1D in nude mice. (N) Bone metastasis BLI of two groups of nude mice, DU145-NC and DU145 Sh-JARID1D, through intracardiac injection. (O) H&E and TRAP staining of tibia of hind limbs of nude mice in DU145-NC and DU145 Sh-JARID1D groups. Scale bars, 200, 50 (H&E), and 20 μm (TRAP).

Inflammatory cytokine levels indicated that the knockdown of JARID1D in DU145 cells led to significant alterations in the levels of 27 inflammatory cytokines. Notably, IL-6, IL-8, TNF-α, and VEGF levels were significantly upregulated in DU145 Sh-JARID1D cells (Figure 4A), and these cytokines showed a pronounced positive correlation with bone metastasis in samples from patients with PCa 25-28. However, no significant differences were observed between DU145 cells expressing WT JARID1D and those expressing catalytically inactive mutants (Figure 4A, right panel). However, in 22RV1 cells, this did not result in the enrichment of cytokines typically associated with bone metastasis (Figure 4A). These findings suggest that JARID1D influences PCa bone metastasis in a manner independent of its enzymatic activity.

RANKL is a classical signaling pathway that induces bone metastasis and promotes the differentiation and activation of osteoclasts, thus accelerating the bone resorption process 29. Therefore, we investigated the effect of JARID1D on RANKL expression. RT-PCR and ELISA revealed a significant increase in RANKL levels following JARID1D knockdown in DU145 cells (Figures 4B and C), no change was observed in 22RV1 cells (Figures S2G and H). These findings indicate that JARID1D knockdown in DU145 cells may promote bone metastasis.

To test this hypothesis, we established an *in vitro* bone cell culture system and an *in vivo* bone metastasis model. *In vitro* studies, bone marrow cells from C57BL/6J mice were induced with 100 ng/mL RANKL and 20 ng/mL macrophage colony-stimulating factor (M-CSF) for 3 d. After the initial induction, the conditioned media for both the NC and Sh-JARID1D treated groups were refreshed, and the cultures were continued for an additional 3 d (Figure 4D). On the 6th d of culture, the TRAP assay indicated that only the conditioned medium from DU145 Sh-JARID1D cells was able supported osteoclast maturation, demonstrating an induction efficacy comparable to that of the positive control (continuously stimulated with RANKL and M-CSF). (Figure 4E, Figure S2I). Similar observations were made with osteoclasts derived from RAW264.7, suggesting a consistent pattern across various models (Figure 4G, Figure S2K).

In the subsequent RT-PCR experiments, the group induced with conditioned medium from DU145 Sh-JARID1D cells showed a significant upregulation of the majority of genes associated with osteoclast differentiation (Figures 4F and H). In contrast, the group induced with conditioned medium from 22RV1 cells showed no significant differences (Figures S2J and L). These findings highlighted the distinct effects of JARID1D-mediated cell origin-specific conditioned media on the molecular mechanisms underlying osteoclast differentiation.

Through* in vivo* experiments, we established a mouse model of bone metastasis by intracardially injecting different PCa cell lines, including 22RV1-NC, 22RV1 Sh-JARID1D, DU145-NC, and DU145 Sh-JARID1D. The results showed distinct metastatic signals in nude mice in the 22RV1 Sh-JARID1D group, which were predominantly localized to the lungs without evident bone diffusion or colonization (Figure S2M). Radiography and CT scans failed to detect any “bone etching” phenomenon (Figure S2N-Q). However, the DU145 Sh-JARID1D group exhibited intense and widespread metastatic signals, predominantly affecting bones, including the skull, mandible, forelimb, hindlimb, and spine (Figure 4I, Figure S2A). Micro-CT analysis confirmed significant bone erosion in the tibia of the hind limbs and radius of the forelimbs (Figure 4J, Figure S2B). A marked decrease in bone density, resulting in a more porous appearance, was observed at the cross-sectional end of the long shaft (Figure 4J, Figure S2C). Three-dimensional (3D) reconstruction and quantitative analysis of micro-CT scans in DU145 Sh-JARID1D mice indicated an enlargement of the osteolytic zone at the bone metastasis site (Figure 4K). Conversely, a significant reduction in the relative bone volume and trabecular thickness was observed (Figure 4L). Changes in the forelimb radius corroborate these findings (Figures S2D-F). Furthermore, compared to the control group, the metastatic ability of nude mice in the DU145 Sh-JARID1D group was weaker, and their survival time was shorter (Figures 4M and N). Histological examination revealed a significant increase in the number of tumor cells and TRAP^+^ osteoclasts on the bone surface at the bone metastasis site in the DU145 Sh-JARID1D group (Figure 4O).

Next, we investigated whether JARID1D influences the osteoblastic characteristics of bone metastases in our model, because DU145 cells exhibit a mixed osteolytic/osteoblastic phenotype in bone metastases 30-31. Our findings revealed that in Sh-JARID1D tumor-associated bone cells, osteocalcin expression was increased compared to that in the control group, which is indicative of osteoblasts and the bone formation process, suggesting an enhanced trend in osteoblast differentiation (Figure S3A). In addition, we observed a general reduction in the expression of osteoblastic metastasis markers, including OPG, PGK1, substance P, and EMID1, in Sh-JARID1D bone metastases relative to that in the control group (Figures S3A and B). Similarly, in a co-culture system using DU145 conditioned medium and mouse embryonic osteoblast MC3T3-E1 subclone 14 cells, we confirmed that the knockdown of JARID1D stimulated the differentiation and mineralization of osteoblasts (Figures S3C and D). Collectively, these results indicate that the absence of JARID1D modulates the activities of both osteoclasts and osteoblasts, affects bone resorption and bone remodeling triggered by osteolytic activity, and consequently promotes the progression of PCa bone metastases.

### JARID1D H3K4 modification dynamically regulates AR transcription

**Figure 5:**
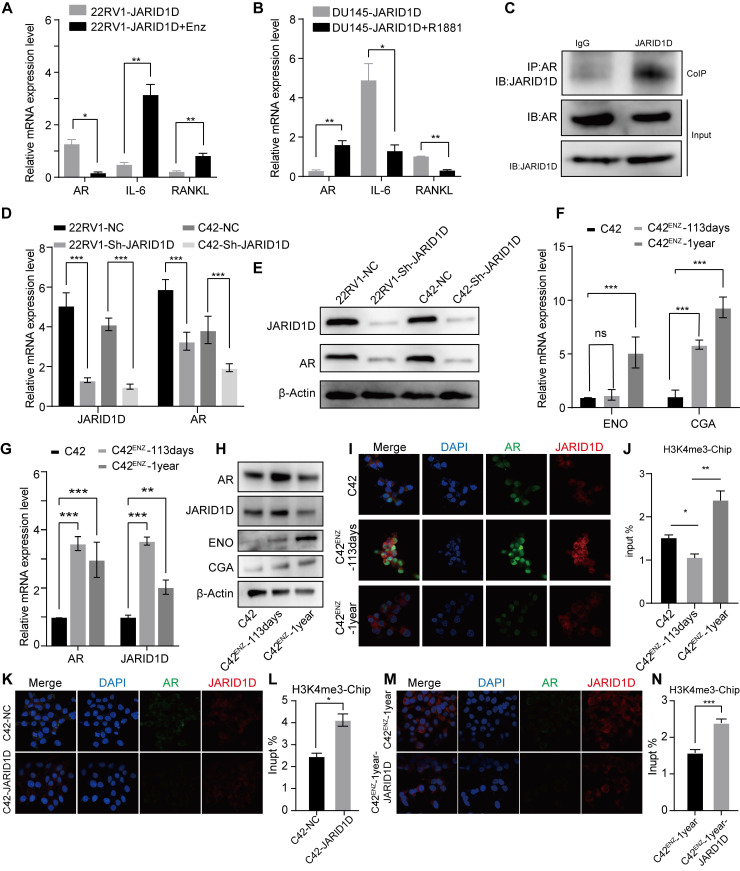
** H3K4 modification by JARID1D dynamically regulates AR transcription.** (A) Quantitative RT-PCR was used to analyze AR, IL-6, and RANKL expression levels in 22RV1 Sh-JARID1D cells treated with Enz (B) Quantitative RT-PCR was used to analyze AR, IL-6, and RANKL expression levels in DU145 Sh-JARID1D cells treated with R1881. (C) Before protein blotting with mutual antibodies, anti-JARID1D, anti-AR, or control antibodies were used to perform Co-IP on 22RV1 cells grown in serum-containing medium;. (D) RT-PCR analysis of AR and JARID1D mRNA expression levels after transfection with si-RNA in 22RV1 and C42 cells. (E) Western blotting of AR and JARID1D protein expression levels after transfection with siRNA in 22RV1 and C42 cells; (F) mRNA expression level of NSE and CGA. (I, K, and M) Co-localization and expression changes of JARID1D and AR. JARID1D is labeled with red fluorescence, AR is labeled with green fluorescence and the cell nucleus is counterstained with DAPI (blue). (J, L, and N) Histone methylation levels at AR enhancer regions.

JARID1D knockdown does not enhance bone metastasis in AR-dependent CRPC cells (22RV1); however, bone metastasis is enhanced in AR-independent NEPC cells (DU145). Therefore, we hypothesized that this discrepancy may be attributed to the dynamic regulation of AR. To test this hypothesis, we selected 22RV1 Sh-JARID1D cells and further reduced the AR levels by treatment with the AR antagonist ENZ. Our findings indicate a significant increase in RANKL levels in these cells, implying a high potential for bone metastasis (Figure 5A).

In addition, we consistently treated AR-positive C42 cells with ENZ for 1 year. Western blotting revealed a significant reduction in AR expression following this treatment (Figure S4A). Subsequently, we injected ENZ-treated C42 cells into the tibial marrow cavity of mice and administered a daily oral dose of 25 mg/kg ENZ to nude mice to progressively deplete the AR. After a 20-d period, in vivo imaging of small animals demonstrated a marked increase in metastatic signals in the ENZ intragastric treatment group (Figure S4B), suggesting a potential role for AR in the regulation of bone metastasis in PCa.

In contrast, in DU145 Sh-JARID1D cells, which effectively promoted bone metastasis, an increase in AR levels was induced by treatment with the AR agonist R1881, thereby leading to a significant downregulation of RANKL levels (Figure 5B). Thus, it is plausible to consider that the depletion or near-depletion of AR signaling may be highly conducive to PCa bone metastasis. This observation aligns with previous clinical findings. The loss of AR signaling often triggers metastasis in patients with late-stage PCa, and the progression of CRPC to NEPC is primarily due to alterations in AR expression and dysregulation of AR signal transduction 32-33. Therefore, exploring the relationship between JARID1D and the AR is crucial for assessing the development of PCa metastasis.

JARID1D directly modulated AR expression in CRPC cell lines (Figure 5C). When JARID1D was downregulated, AR expression correspondingly decreased (Figures 5D and E), indicating that JARID1D serves as a molecular mediator of AR depletion and is positioned upstream in the regulatory network that influences AR levels. Furthermore, we explored the changes in the binding affinity between JARID1D and the AR throughout the transition from CRPC to NEPC. Some CRPC cells gradually develop resistance to ENZ and may transform into NEPC 34.

We treated C42 cells from a CRPC model with 20 μM ENZ for varying durations, resulting in C42^ENZ^-113 (113 d) and C42^ENZ^-1-year groups. During this treatment, the expression of NEPC markers, including ENO and CGA, progressively increased (Figures 5F and G). Throughout this transition, JARID1D and AR levels initially increased and then decreased (Figure 5H). The IF results indicated that the binding affinity between JARID1D and AR underwent dynamic changes (Figure 5I). As JARID1D is known to demethylate H3K4me3 marks 15, we investigated whether the affinity between JARID1D and AR regulates the level of H3K4me3 in the AR promoter region. ChIP data revealed that the enhanced affinity between JARID1D and AR correlated with reduced H3K4me3 levels. Conversely, diminished affinity was associated with increased H3K4me3 levels (Figure 5J).

As depicted in Figures 5K-N, the knockdown of JARID1D in the C42 and C42^ENZ^-1-year cell lines led to a significant decrease in AR expression, reduction in binding affinity, and an increase in H3K4me3 levels compared to the control group. Notably noteworthy that the C42^ENZ^-1-year cells showed a diminished capacity for H3K4me3 demethylation following JARID1D knockdown relative to C42 cells (Figures 5L and N). This finding suggests potential differences in the epigenetic mechanisms involved, which may play a role in AR transcriptional dysregulation.

### JARID1D regulates the vicious cycle of PCa bone metastasis through the AR-MAOA signaling pathway

**Figure 6:**
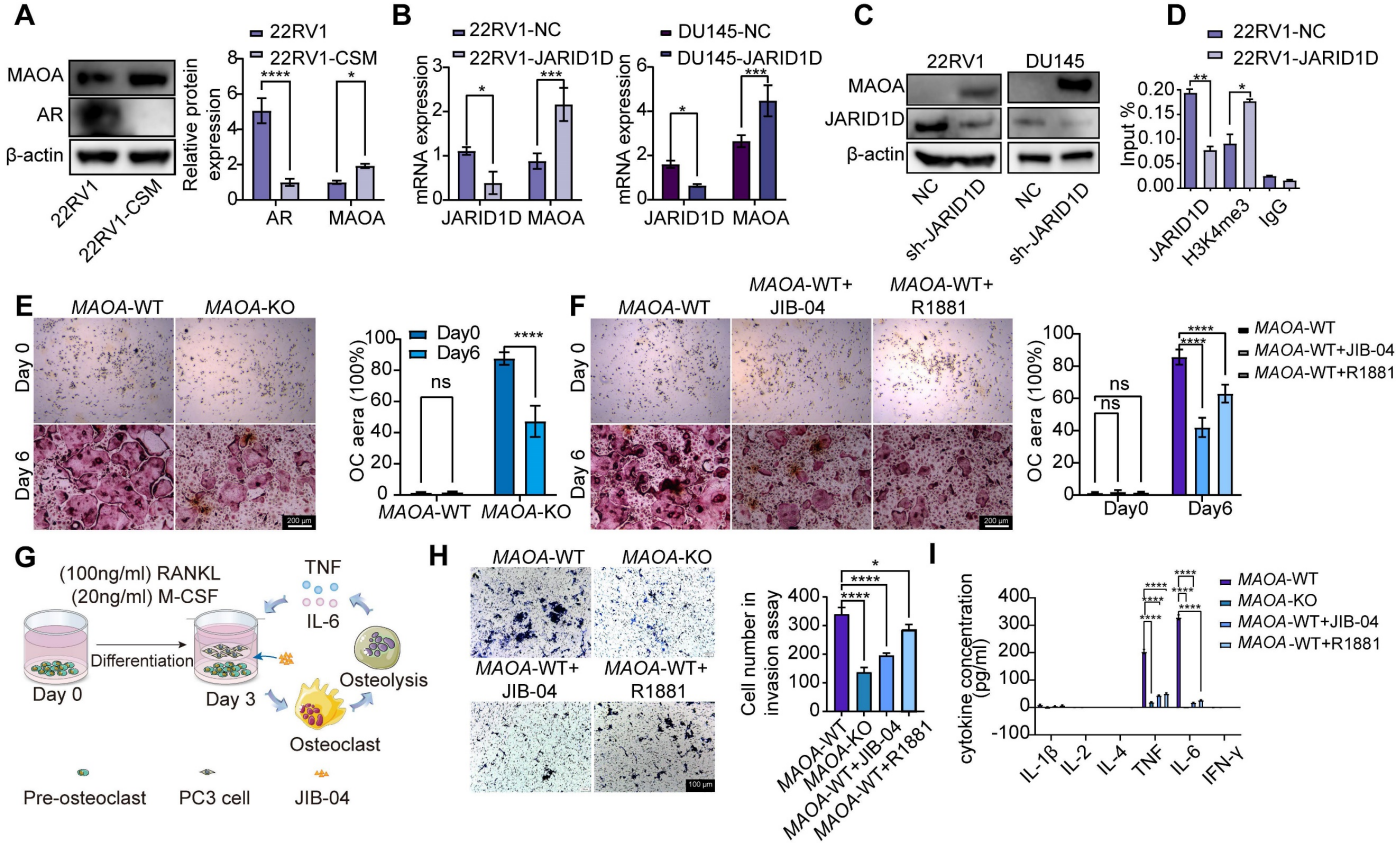
** JARID1D regulates the vicious cycle of PCa bone metastasis through the AR-MAOA signaling pathway.** (A) Western blot analysis of AR and MAOA protein expression levels after treat with an androgen-deprivation medium in 22RV1 cells. (B) mRNA expression levels of MAOA after knockdown of JARID1D in 22RV1 and DU145 cells. (C) mRNA expression levels of MAOA after knockdown of JARID1D in 22RV1 and DU145 cells; (D) ChiP-PCR was used to analyze effect of knocking down JARID1D in 22RV1 cells on H3K4me3 binding level at MAOA promoter binding region; (E) TRAP staining and quantification results of osteoclasts induced from *MAOA*-WT and *MAOA*-KO mice on days 0 and 6 (F) Representative images and quantification results of TRAP staining for different treatment groups on days 0 and 6;. (G) Model of co-culture of PC3 cells with pre-osteoclasts. Murine *MAOA*-WT pre-osteoclasts were seeded into the wells of six-well plates. PC3 cells were seeded into Transwell inserts in the six-well plates and treated with JIB-04 (100 μM); (H) Representative images and quantification results of the Transwell from different treatment groups; (I) Multi-factor detection of IL-1β, IL-2, IL-4, IL-6, TNF, and IFN-γ content in the culture supernatant of different treatment groups.

The current challenge is to elucidate the downstream mechanisms of the JARID1D-mediated demethylation of AR. Previous studies, including ours 35, suggested that the AR signaling pathway can regulate the MAOA 36. In an androgen-deficient context, AR can initiate MAOA expression, thus enabling the transition of androgen-dependent LNCaP cells to an androgen-independent growth pattern 37,38. Our results are consistent with this, showing that culturing 22RV1 cells in androgen-free medium (charcoal-stripped medium [CSM]) resulted in decreased AR expression and increased MAOA levels (Figure 6A). Moreover, when JARID1D was knocked down in 22RV1 and DU145 cells, MAOA expression was significantly upregulated (Figures 6B and C). ChIP assays further revealed that JARID1D knockdown increased the occupancy of H3K4me3 at the MAOA promoter (Figure 6D). These findings indicate that MAOA may serve as a downstream effector in the JARID1D-AR signaling axis, thereby driving PCa metastasis to the skeletal system.

To confirm the role of MAOA in bone metastasis, we isolated bone marrow cells from both *MAOA*-WT and *MAOA*-KO mice and continuously induced them with 100 ng/mL RANKL and 20 ng/mL M-CSF. After 6 d, TRAP staining revealed a significant reduction in the capacity for osteoclast differentiation following MAOA knockout (Figure 6E). This suggests that MAOA promoted osteoclast differentiation and facilitated bone metastasis.

Furthermore, on the 3rd d of osteoclast differentiation from WT cells, the introduction of the JARID1D agonist JIB-04 and AR agonist R1881 significantly attenuated the osteoclast differentiation process (Figure 6F). These findings further substantiate the importance of the JARID1D-AR-MAOA signaling pathway in PCa bone metastasis.

During bone metastasis, the activated osteoclasts degrade the bone matrix and release multiple cytokines. These cytokines further stimulate the growth and metastasis of tumor cells, establishing a vicious cycle that exacerbates the progression of bone metastasis 29. To mimic this cycle within the bone microenvironment of PCa, we co-cultured osteoclasts induced for 3 d from *MAOA*-WT mice with PC3 cells; JIB-04 and R1881 were also added (Figure 6G). The Transwell assay revealed that the invasive capacity of PC3 cells was markedly inhibited by MAOA knockout and agonist treatment (Figure 6H). Moreover, the multi-factor detection results indicated that the ability of osteoclasts to secrete TNF and IL-6 were reduced (Figure 6I). In summary, our findings indicate that a targeted therapeutic strategy focusing on the JARID1D-AR-MAOA axis can effectively disrupt this cycle, providing a novel therapeutic approach for the management of bone metastasis in PCa.

### JARID1D directly mediates osteoclast differentiation through JunD, inhibiting PCa bone metastasis

**Figure 7:**
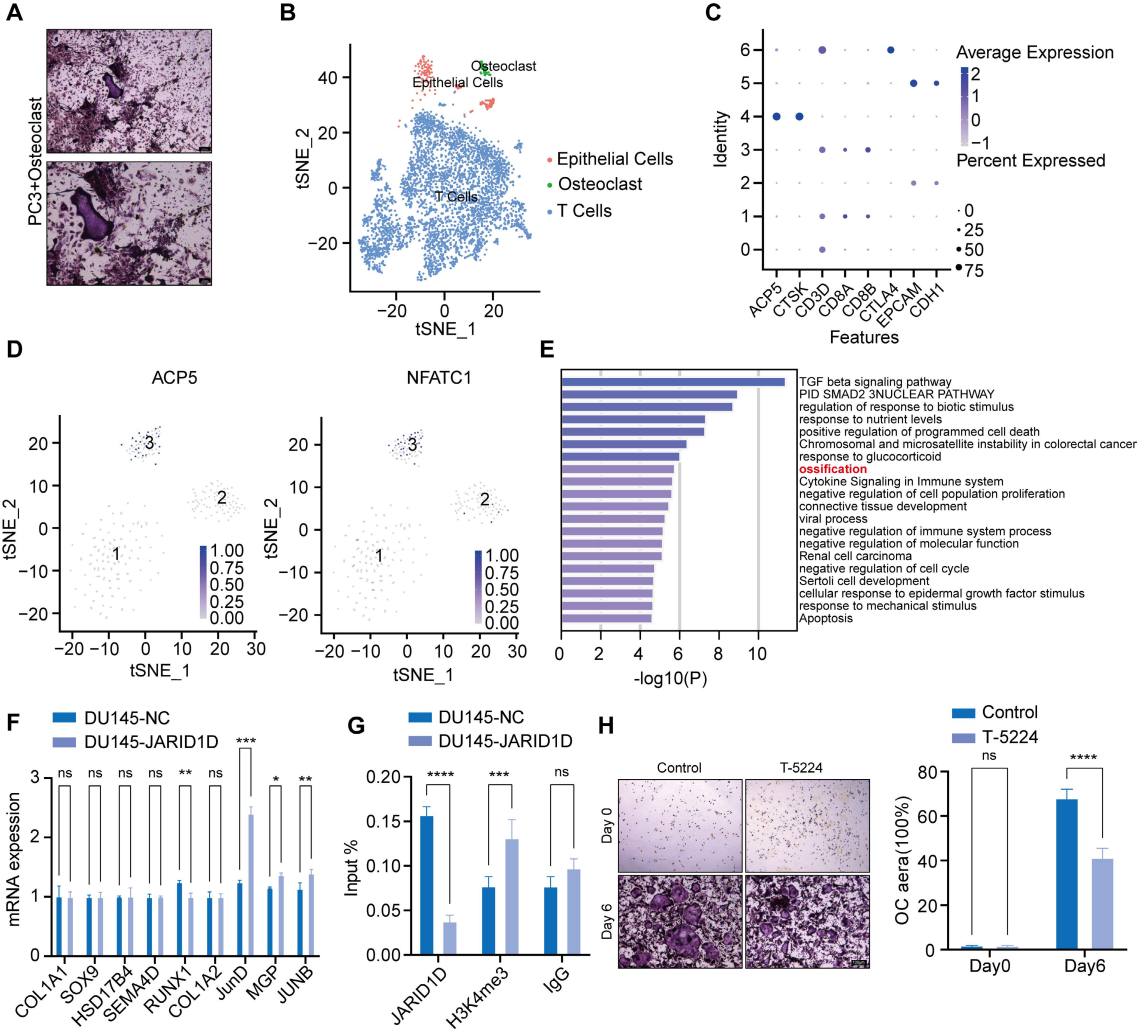
** JARID1D directly mediates osteoclast differentiation through JunD, inhibiting PCa bone metastasis.** (A) TRAP staining of osteoclast co-cultured with PC3 (scale bars, 200 and 100 μm); (B-C) Single-cell omics data of human tumor bone metastases reveal three cell types; (D) Single-cell omics data from human tumor bone metastases show the detection of osteoclast-specific NFATC1 and ACP5 mRNA in tumor epithelial cell populations; (E) Gene Ontology (GO) Enrichment Analysis for TAOC and OC Groups; (F) RT-PCR detection of the expression of ossification-related genes after knockdown of JARID1D in DU145 cells; (G) ChiP-PCR was used to analyze effect of knocking down JARID1D in DU145 cells on H3K4me3 binding level at JUND promoter binding region; (H) Representative images and quantification results of TRAP staining for the control group and the T-5224 treatment group on days 0 and 6.

Recent research has uncovered a new subtype within tumors known as tumor-associated osteoclasts (TAOC), which can directly interact with osteoclasts and influence their functionality 39. Based on this theory, PC3 cells were placed at the center of a culture dish containing osteoclasts that had been induced for 3 d. TRAP staining revealed that osteoclasts near the PC3 cells demonstrated a robust capacity for differentiation (Figure 7A). This finding suggests that tumor cells directly influence osteoclast activity and bone metastasis in PCa.

To investigate these mechanisms, we categorized single-cell sequencing datasets (GSE143791) from clinical patients with PCa bone metastasis into three major subpopulations, tumor epithelial cells, osteoclasts, and immune cells, based on specific marker genes (Figures 7B and C). Subsequently, within the tumor epithelial cell group, we successfully identified a cluster with high expression of ACP5 and NFATC1, which we defined as the TAOC population (Figure 7D, Figure S5A) 39. Differential gene enrichment analysis of TAOC and osteoclasts revealed that nine genes were closely related to bone differentiation (Figure 7E). Notably, among these genes, the knockdown of JARID1D led to the most significant changes in JunD expression, suggesting that JARID1D targets JunD to modulate osteoclast differentiation (Figure 7F). Western blot analysis confirmed these findings, demonstrating an increase in JunD expression following the JARID1D knockout (Figure S5B). Moreover, osteoclast differentiation capacity was reduced in the group treated with the JunD inhibitor (T-5224) (Figure 7H, Figure S5C). The ChIP assay results further indicated that JARID1D knockdown increased H3K4me3 levels at the JunD promoter (Figure 7G), suggesting that JARID1D in tumor cells can directly demethylate and regulate JunD in osteoclasts, thus affecting bone metastasis in PCa.

### JIB-04 impeded bone metastasis of PCa and prolonged survival by disrupting the AR-MAOA and JunD signal network

Suppression of JARID1D enhanced bone metastasis in PCa, offering a theoretical foundation for the therapeutic application of JARID1D agonists. Therefore, the JARID1D agonist-JIB-04 was selected to confirm the function of JARID1D. First, JIB-04 inhibited the proliferation of DU145, and the IC50 was approximately 78.25 μm (Figure S6A). To clarify the effect of JIB-04 on the expression of JARID1D in PCa cells, DU145 cells were treated with 50 μm JIB-04. The results showed that JIB-04 restored the JARID1D knockdown in DU145 cells (Figures S6B and C) and acts as an agonist of JARID1D.

**Figure 8:**
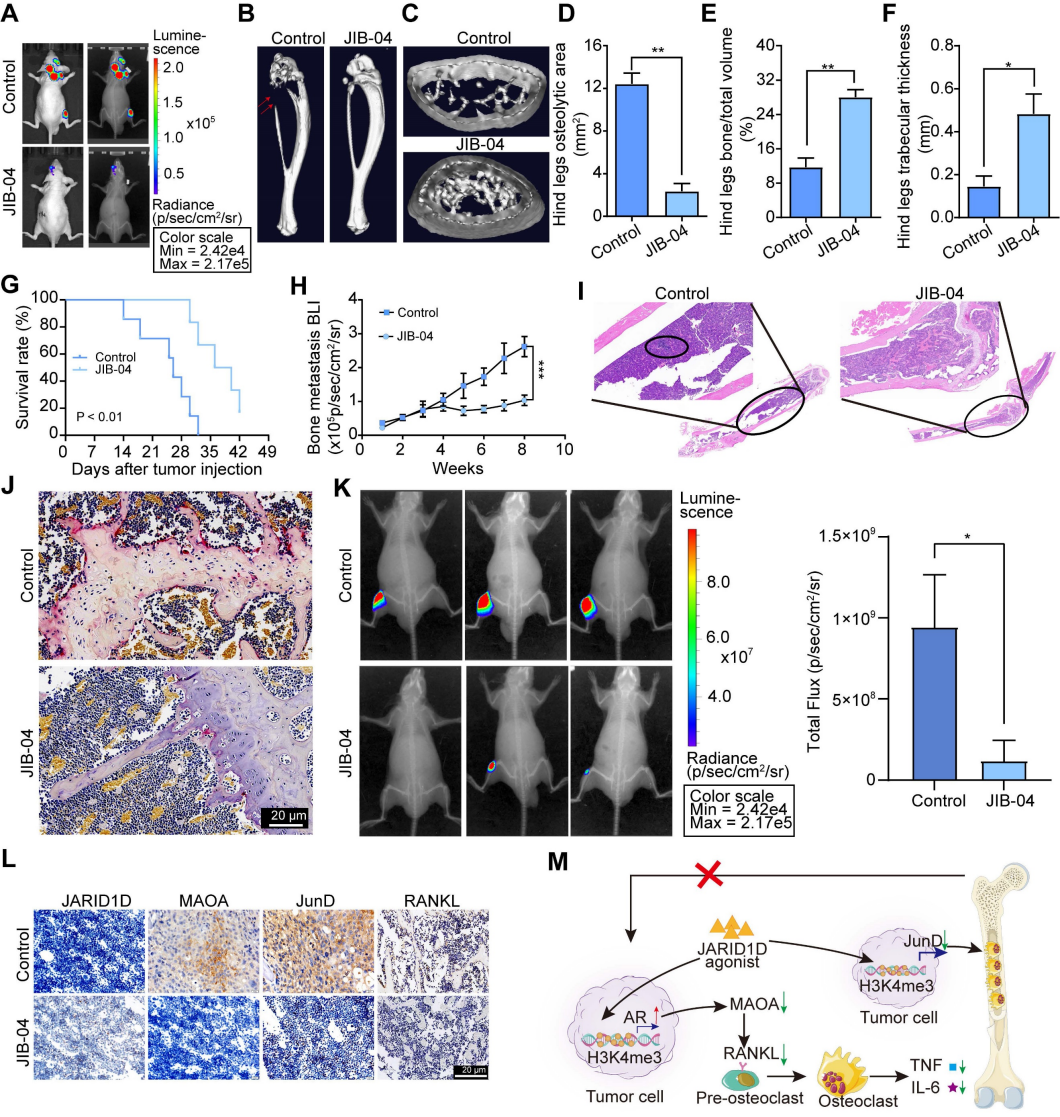
** JIB-04 impeded bone metastasis of PCa and prolonged survival by disrupting the AR-MAOA and JunD signal network.** (A) Bioluminescence and radiography images of nude mice after JIB-04 treatment; (B) Micro-CT image of tibia of hindlimb of representative nude mouse after JIB-04 treatment; (C) Cross-section of tibia of hindlimb of representative nude mouse after JIB-04 treatment; (D-F) Quantitative map of osteolytic area (D), relative bone volume (E), and trabecular thickness (F) of tibia of hindlimb in (B, C) above (n = 3); (G) Survival curves of nude mouse after JIB-04 treatment; (H) Bone metastasis BLI of nude mouse after JIB-04 treatment; (I) H&E staining of tibia of hind limbs of nude mice after JIB-04 treatment. Scale bars, 200 and 50 μm; (J) TRAP staining of tibia of hind limbs of nude mice after JIB-04 treatment. Scale bars, 50 μm; (K) Radiography images of nude mice after JIB-04 treatment (n=3); (L) IHC analysis of expression levels of JARID1D, MAOA, JunD and RANKL in tibia of hind limbs of nude mice after JIB-04 treatment. Scale bars, 50 μm. (M) The schematic illustrates the interactions between tumor cells expressing JARID1D and the bone microenvironment.

To determine whether JIB-04 mitigates bone metastasis *in vivo*, we developed a bone metastasis model of PCa by intracardially injecting DU145 Sh-JARID1D cells into nude mice. At the 2-week mark, JIB-04 was administered intraperitoneally to the mice. Radiographic assessments revealed numerous metastases to the skull, mandible, forelimb, spine, and hind limb bones of nude mice in the control group (Figure 8A, Figure S6D). Notably, JIB-04 treatment significantly reduced bone metastasis (Figure. 8A, Figure S6D). Micro-CT analyses indicated that osteolytic lesions in both the hind limb tibia and forelimb radius were marginally more pronounced in the JIB-04 treatment group than in the control group (Figures 8B and C, Figures S6E and F). Quantitative analysis further revealed that JIB-04 treatment led to a reduction in the osteolytic zone area, along with a significant enhancement in the relative bone volume and trabecular thickness (Figures 8D-E, Figures S6G-I). Simultaneously, JIB-04 treatment extended the survival period of nude mice with bone metastasis (Figure 8G) and further decreased the intensity of Bioluminescence Imaging (BLI) (Figure 8H). Histological examination revealed that JIB-04 administration significantly reduced the presence of tumor cells and TRAP^+^ osteoclasts on the bone surface at metastatic sites in nude mice (Figures 8I and J). In addition, we observed a concurrent decrease in the expression of osteocalcin and osteoblastic metastatic markers (Figure S7A), consistent with the in vitro findings that demonstrated diminished differentiation and mineralization capabilities in mouse embryonic osteoblast MC3T3-E1 subclone 14 cells following treatment with JIB-04 (Figures S7B and C).

Moreover, we directly inoculated PC3 cells, which are predisposed to bone metastasis and lack JARID1D expression 16, into the medullary cavity of the tibia of nude mice, creating a PBS control group and JIB-04 treatment group. Beginning on the 2nd d after model establishment, we administered intraperitoneal injections of PBS and JIB-04 twice daily for 20 d. Radiological assessments indicated that the JIB-04 treatment group exhibited slower tumor growth (Figure 8K). Ultimately, our investigation into JARID1D-mediated signaling within the tumor-bone cell interaction revealed that following JIB-04 treatment, there was upregulation of JARID1D, whereas MAOA and JunD expression were downregulated. This finding offers favorable evidence for targeted therapy of JARID1D (Figure 8L).

In summary, our study revealed that a deficiency in JARID1D expression initiates a detrimental feedback loop between tumor cells and osteoclasts, which contributes to the progression of PCa metastasis to the skeletal system and visceral organs. The use of a JARID1D agonist has shown the potential to significantly counteract this adverse cycle, presenting a promising therapeutic strategy (Figure 8M).

## Discussion

Bone is the predominant form of metastasis in patients with PCa. Further research has implicated JARID1D in the metastatic cascade of various cancers 16,40,41. However, the specific impact of JARID1D on the metastatic behavior of PCa and the broader implications of its epigenetic regulation are yet to be fully elucidated. Here, we showed that JARID1D levels were diminished in metastatic PCa relative to the primary tumor, with an even more pronounced reduction observed in bone metastatic PCa (Figure 1H). In addition, patients with PCa exhibiting diminished JARID1D expression correlated with reduced survival duration and poorer prognosis (Figures 1B and C). These findings highlight the potential of JARID1D as a prognostic biomarker that may contribute to the inhibition or slowing of PCa progression.

High heterogeneity is an important clinical feature of PCa. Hormone-sensitive PCa typically progresses to CRPC after treatment, until NEPC, an aggressive variant of PCa, develops 42,43. To explore the role of JARID1D in PCa progression, we selected CRPC (22RV1) and NEPC (DU145) cell lines to knockdown JARID1D expression. The results showed that suppression of JARID1D expression and alteration of its enzymatic activity in both cell lines significantly increased the cells' *in vitro* invasiveness and their capacity for *in vivo* metastasis, particularly to the lungs (Figures 2 and 3). We established a mouse model of bone metastasis by intracardiac injection and assessed the effect of JARID1D on bone metastasis by inducing osteoclast and osteoblast differentiation in vitro. We observed that the reduction in JARID1D expression in CRPC 22RV1 cells (AR-positive) did not promote osteoclast differentiation or bone metastasis (Figures S2I-R). Conversely, the downregulation of JARID1D in NEPC DU145 cells (AR-negative) significantly enhanced osteoclast and osteoblast differentiation and the bone metastatic potential of PCa (Figures 4E-O, Figures S2A-C S3A-D). This indicates that the disparity in outcomes is primarily attributable to the dysregulation of AR dynamics at various stages of PCa progression.

The transition from CRPC to NEPC is associated with the gradual failure of the AR signaling pathway, leading to the acquisition of new biological characteristics and aggressiveness of tumor cells. Downregulation or loss of function of AR may enhance the interaction between tumor cells and the bone microenvironment, promoting bone metastasis 44-46. To confirm whether bone metastasis was caused by AR depletion, AR-positive 22RV1-JARID1D cells were treated with the AR inhibitor ENZ to further reduce AR levels. This treatment elicited alterations in cells favorable for bone metastasis, as evidenced by a marked increase in RANKL levels (Figure 5A). Conversely, in AR-negative DU145-JARID1D cells, treatment with the AR agonist R1881 led to a significant decrease in initially elevated RANKL levels (Figure 5B). Collectively, these findings suggest that the knockdown of JARID1D and consequent AR depletion significantly promotes bone metastasis.

JARID1D is capable of demethylating H3K4me3 marks to inhibit tumor invasion 15; however, whether the affinity between JARID1D and AR regulates the level of H3K4me3 in the AR promoter region needs further investigation. Our ChIP results demonstrated that the H3K4me3 mark, catalyzed by JARID1D, mediates the dynamic regulation of AR within its promoter-binding region during the progression from CRPC to NEPC (Figure 5I). The enzymatic activity of JARID1D in catalyzing H3K4me3 was significantly reduced during the NEPC phase compared to that during the CRPC phase. This disparity in epigenetic regulation may contribute to the gradual exhaustion of AR, leading to the development of bone metastasis (Figures 5K-N). MAOA synergizes with AR through reciprocal crosstalk to amplify AR-directed PCa disease progression, including the aggressive castration-resistant variant 36. Our results revealed that reduced AR function leads to increased MAOA expression, which enhances osteoclast differentiation. Moreover, the upregulation of MAOA expression was regulated by JARID1D through H3K4me3 modification (Figure 6). Our findings underscore the critical role of JARID1D in modulating the AR/MAOA signaling axis, leading to PCa bone metastasis. Decreased JARID1D expression in tumor cells correlated with reduced AR activity, increased MAOA levels, and elevated RANKL secretion. These interrelated events synergize to stimulate osteoclast differentiation, trigger bone resorption, and facilitate the release of TNF and IL-6, thereby promoting tumor metastasis.

Our subsequent studies demonstrated that the targeted ablation of MAOA coupled with the administration of agonists specific to JARID1D or AR effectively interrupted this vicious cycle of the pathological process. Therefore, we propose that interventions aimed at the JARID1D-AR-MAOA signaling network may provide a promising strategy for the treatment of PCa bone metastasis.

Recent advancements have suggested that the presence of tumor cells can lead to the formation of TAOC, a new subtype of osteoclasts. These specialized cells interact directly with osteoclasts, exert a significant influence on their differentiation and maturation, and induce bone metastasis 39. Through the analysis of single-cell RNA sequencing data from patients with bone metastases, we successfully identified TAOC and conventional osteoclast subtypes and identified critical differential genes that regulate osteogenic differentiation (Figure 7E). Notably, activating protein-1(AP-1) transcription factor JunD is a key mediator of osteoclast differentiation and is directly regulated by JARID1D. JunD, integral to bone metabolic pathways, engages with pivotal regulators, such as RANKL and MCP-1, modulating the equilibrium between bone resorption and formation 47-48. An apparent reduction in osteoclast differentiation was observed after treatment with the JunD inhibitor T-5224 (Figure 7H). More importantly, the knockdown of JARID1D resulted in an elevated level of histone modification H3K4me3 within the JunD promoter region (Figure 7H). This enrichment of H3K4me3 is indicative of the activation of JunD transcription, potentially representing a novel regulatory mechanism through which tumor cells exert a direct influence on osteoclast activity. Drawing from these findings, our research elucidates another role of JARID1D in PCa bone metastasis of PCa the H3K4me3-JunD signaling pathway. Consequently, JARID1D agonists may target a multitude of molecules and associated pathways, thereby providing a more extensive inhibitory effect on bone metastasis than conventional single-target therapies such as RANKL inhibition.

The histone demethylase Jumonji C domain agonist JIB-04 is a small-molecule targeted drug that has been used to treat several different tumor types 49-50. The IC50 value was nearly three orders of magnitude, indicating better safety. Notably, JIB-04 shows consistent selectivity for cancer cells 51. Our investigation demonstrated that JIB-04 effectively inhibited bone metastasis induced by JARID1D knockdown and prolonged the survival of mice with PCa bone metastasis (Figure 8). Furthermore, JIB-04 diminished the expression levels of key metastatic mediators, including MAOA, JunD, and RANKL, within metastatic tumor tissues while activating JARID1D (Figure 8L).

## Conclusion

In conclusion, epigenetic regulation by the Y chromosome-encoded histone demethylase JARID1D is a pivotal mechanism for regulating bone metastasis in PCa. It inhibited bone metastasis through the dual signaling axes of H3K4me3-AR-MAOA-RANKL and H3K4me3-JunD (Figure 8M). JARID1D agonists have the potential to inhibit both osteoblastic and osteoclastic bone metastases and could offer a novel strategy for managing resistance to androgen deprivation therapy. These agents represent promising therapeutic options for patients with intermediate-to high-risk PCa, particularly those with significantly reduced AR levels.

## Supplementary Material

Supplementary figures and tables.
