# Supplementary material for: JARID1D-dependent androgen receptor and JunD signaling activation of osteoclast differentiation inhibits prostate cancer bone metastasis through demethylating H3K4
Source: Theranostics. 2025 Jan 1;15(4):1320–37. doi: 10.7150/thno.104135 (PMC11729558; doi:10.7150/thno.104135)
Supplement: Supplementary file 1 — Supplementary figures and tables. [file thnov15p1320s1.zip › Supplementary-Meterial/Supplementary-Material.docx]

Supplementary Material

**JARID1D-dependent androgen receptor and JunD signaling activation of osteoclast differentiation inhibits prostate cancer bone metastasis through demethylating H3K4**

Authors’ names: Yaohua Hu, Zhite Zhao, Qinghua Xie, Hui Li, Chenyang Zhang, Xinglin He, Yifan Ma, Caiqin Zhang, Qinlong Li

*Correspondence:

Changhong Shi

Division of Cancer Biology, Laboratory Animal Center, The Fourth Military Medical University, Xi’an, Shaanxi 710032, China

Tel: 86-29-84774787

Fax: 86-29-83242045

Email: [changhong@fmmu.edu.cn](mailto:changhong@fmmu.edu.cn)

Qinlong Li

Department of Pathology, School of Basic Medicine and Xijing Hospital, State Key Laboratory of Cancer Biology, The Fourth Military Medical University, Xi'an, China.
qinlongli@163.com

**Supplementary Figure**


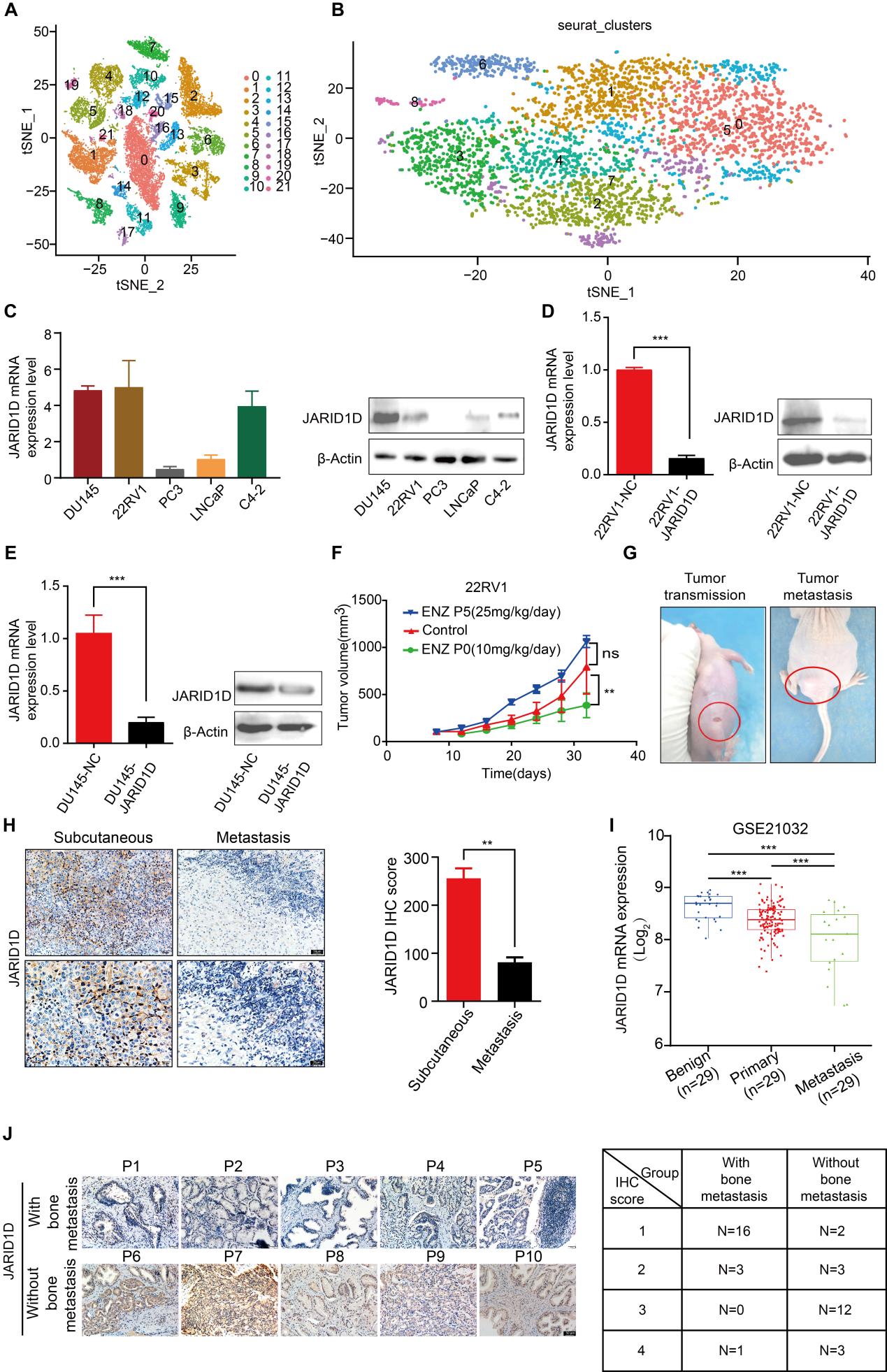


**Supplementary Figure. 1** (A) A t-SNE plot showing 28 cell clusters by unsupervised graph clustering; (B) A t-SNE plot of JARID1D showing normalized expression level in the sub-clusters; (C) Expression levels of JARID1D in prostate cancer cell lines; (D-E) Expression of JARID1D after 22RV1 (D) and DU145 (E) cells was transfected into lentivirus was analyzed using quantitative RT-PCR and western blotting; (F) The tumor growth curves of mice in the Control group, ENZ P0 group and ENZ P5 group; (G) Tumor Metastasis Site Photographs; (H) The expression of JARID1D in subcutaneous and metastatic tumors in mice; (I) JARID1D mRNA levels in PCa tissues were assessed by analyzing the GS21032; (J) IHC analysis of primary prostate cancer samples from 40 patients with and without bone metastasis. Each sample was scored based on the percentage of JARID1D positivity (1 points: 0-25%, 2 points: 26-50%, 3 points: 51-75%, 4 points: 76-100%). Scale bar: 20 μm.


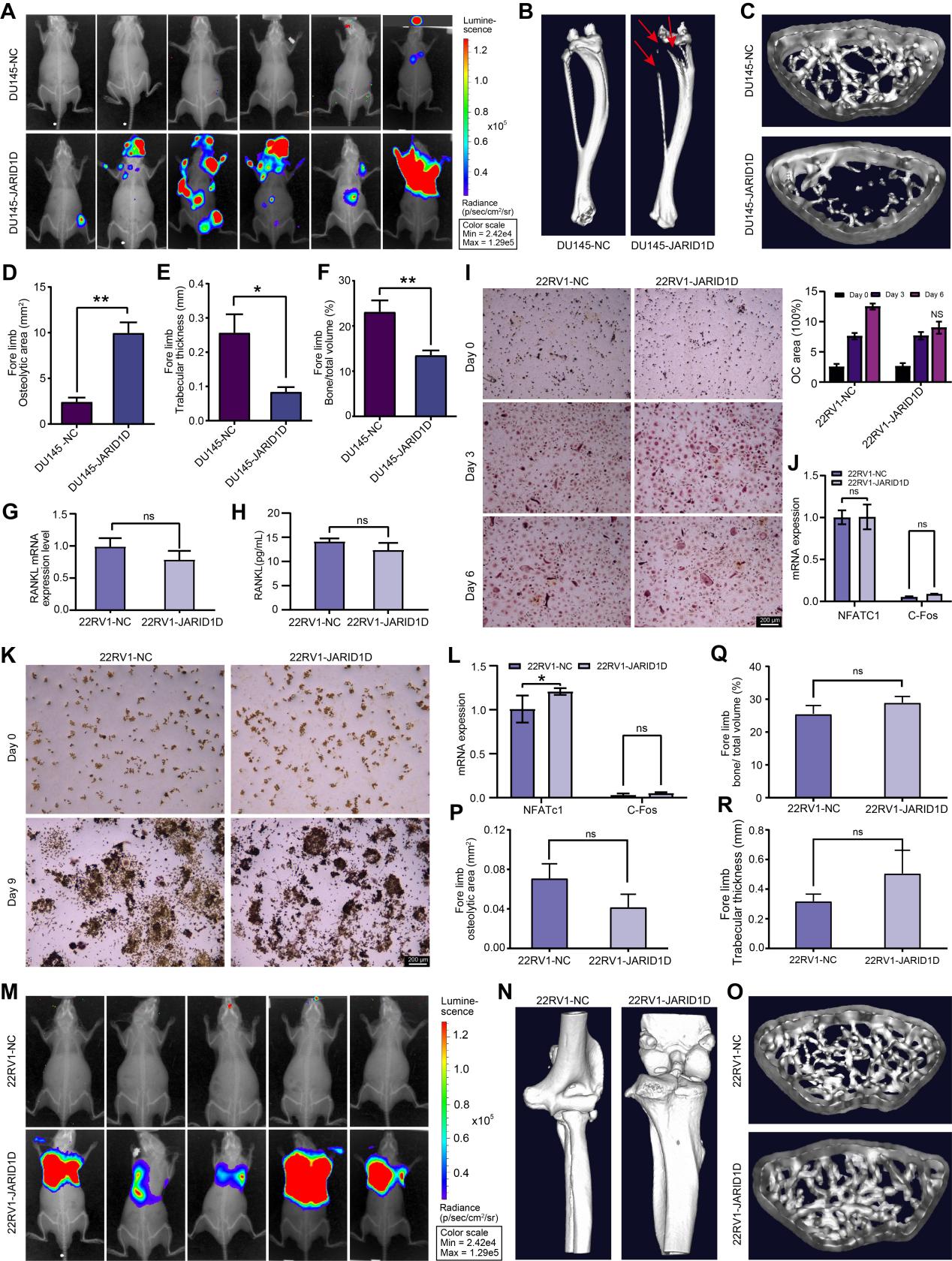


**Supplementary Figure. 2** (A) X-ray image of DU145-NC and DU145 Sh-JARID1D cells intracardially injected into nude mice; (B) Micro-CT image of tibia of hindlimb of representative nude mouse in DU145-NC and DU145 sh-JARID1D groups; (C) Cross section of tibia of hindlimb of representative nude mouse in DU145-NC and DU145 Sh-JARID1D groups; (D-F) Quantitative map of osteolytic area (D) relative bone volume (E) and trabecular thickness (F) of tibia of hindlimb in (A) above (n = 3); (G) RT-PCR detection of RANKL expression in 22RV1 cells with JARID1D knockdown; (H) ELISA detection of RANKL content in the culture supernatant of 22RV1cells after JARID1D knockdown; (I) TRAP staining to assess the differentiation capacity of osteoclasts in different treatment groups and quantification results; (J) RT-PCR analysis of osteoclast differentiation-related gene expression across various treatment groups; (K) Representative TRAP staining images and quantification results of osteoclast differentiation induced by RAW264.7 cell in different treatment groups; (L) RT-PCR analysis of osteoclast differentiation-related gene expression across various treatment groups; (M) X-ray image of 22RV1-NC and 22RV1 Sh-JARID1D cells intracardially injected into nude mice; (N) Micro-CT image of tibia of hindlimb of representative nude mouse in 22RV1-NC and 22RV1Sh-JARID1D groups. (O) Cross section of tibia of hindlimb of representative nude mouse in DU145-NC and DU145 sh-JARID1D groups. (P-R) Quantitative map of osteolytic area (P) relative bone volume (Q) and trabecular thickness (R) of tibia of hindlimb in (M, N) above (n = 3).


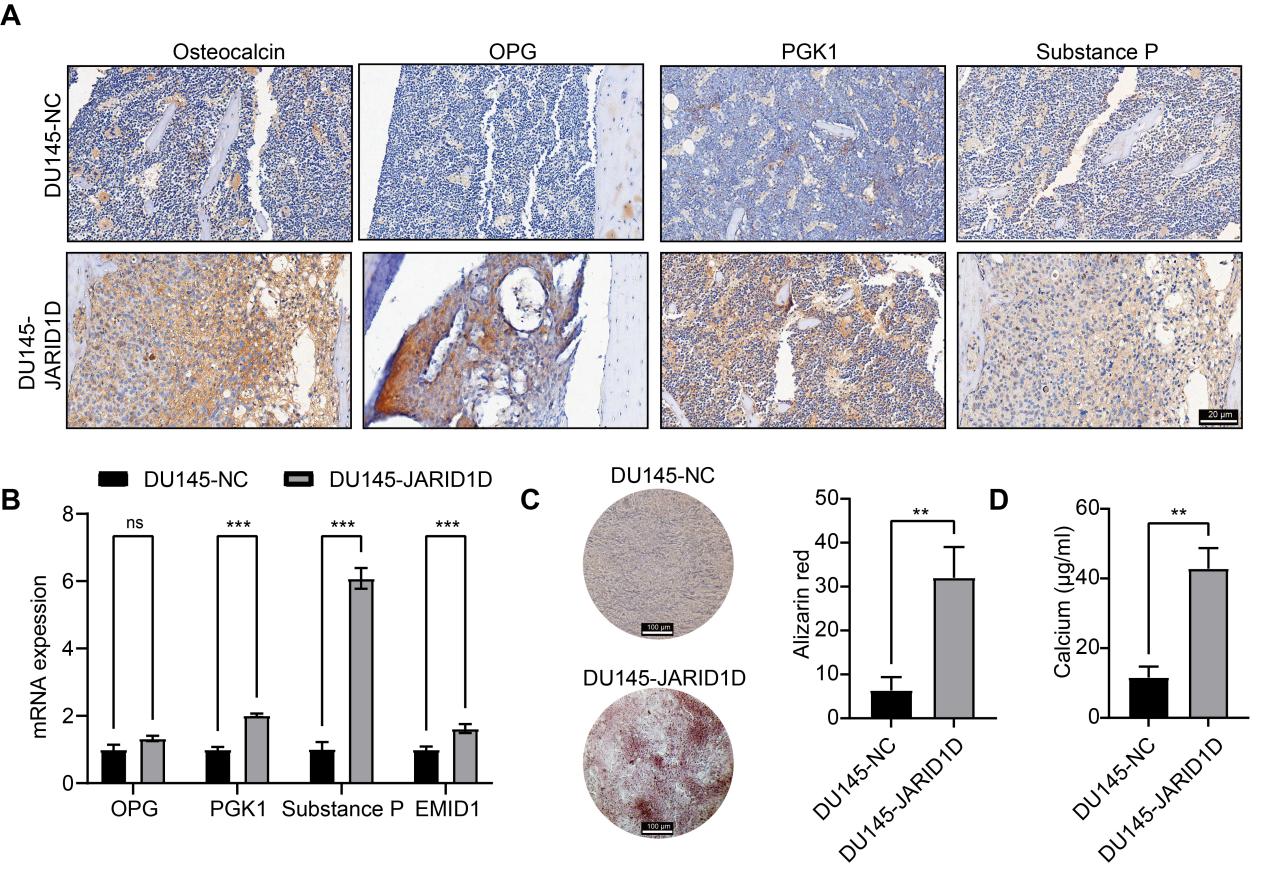


**Supplementary Figure. 3** (A) Representative IHC images of osteocalcin and three reported osteoblastic prostate cancer bone metastasis markers, OPG, PGK1, and Substance P, in tumor-associated osteoblasts from bone metastases in mice inoculated with DU145-NC and DU145-ShJARID1D cells. Scale bar: 20 μm; (B) mRNA expression of 4 reported markers of osteoblastic PCa bone metastases, OPG, PGK1, Substance P and EMID1, in tumor cells by RT-qPCR; (C) mouse embryonic osteoblasts MC3T3-E1 subclone 14 cells were cultured for 16 days in DU145-NC or DU145-ShJARID1D conditioned media, followed by an Alizarin Red S staining assay. The figures show representative wells from each group as well as the relative absorbance of Alizarin Red S; (D) Measurement of calcium content in media from each group (n=3) in (C) .


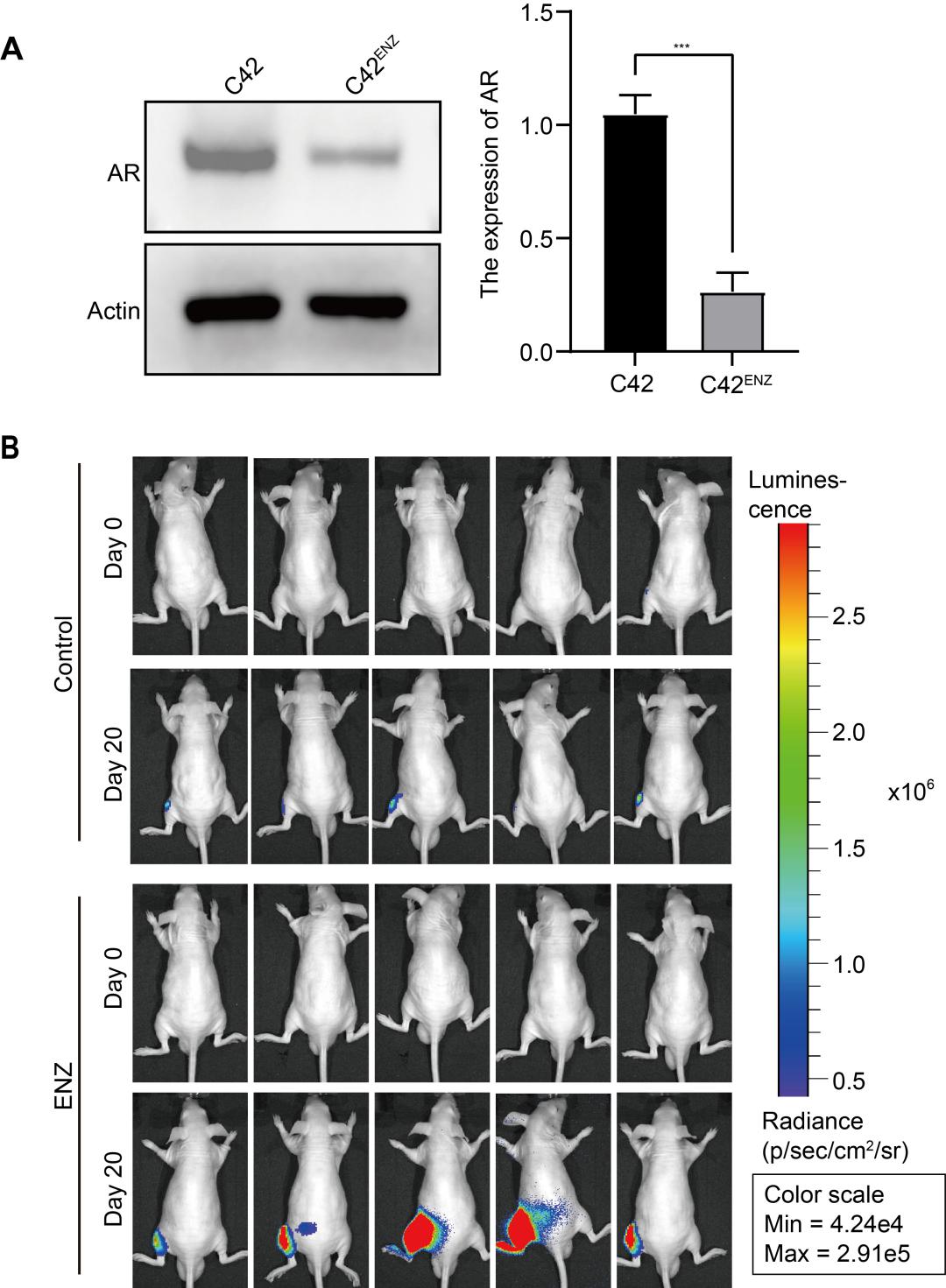


**Supplementary Figure. 4** (A) The protein expression levels of AR in C42 and C42^ENZ^ (induced for one year with 20 μm ENZ); (B) Representative images of in vivo imaging in small animals.from different treatment groups on Day 0 and Day 20.


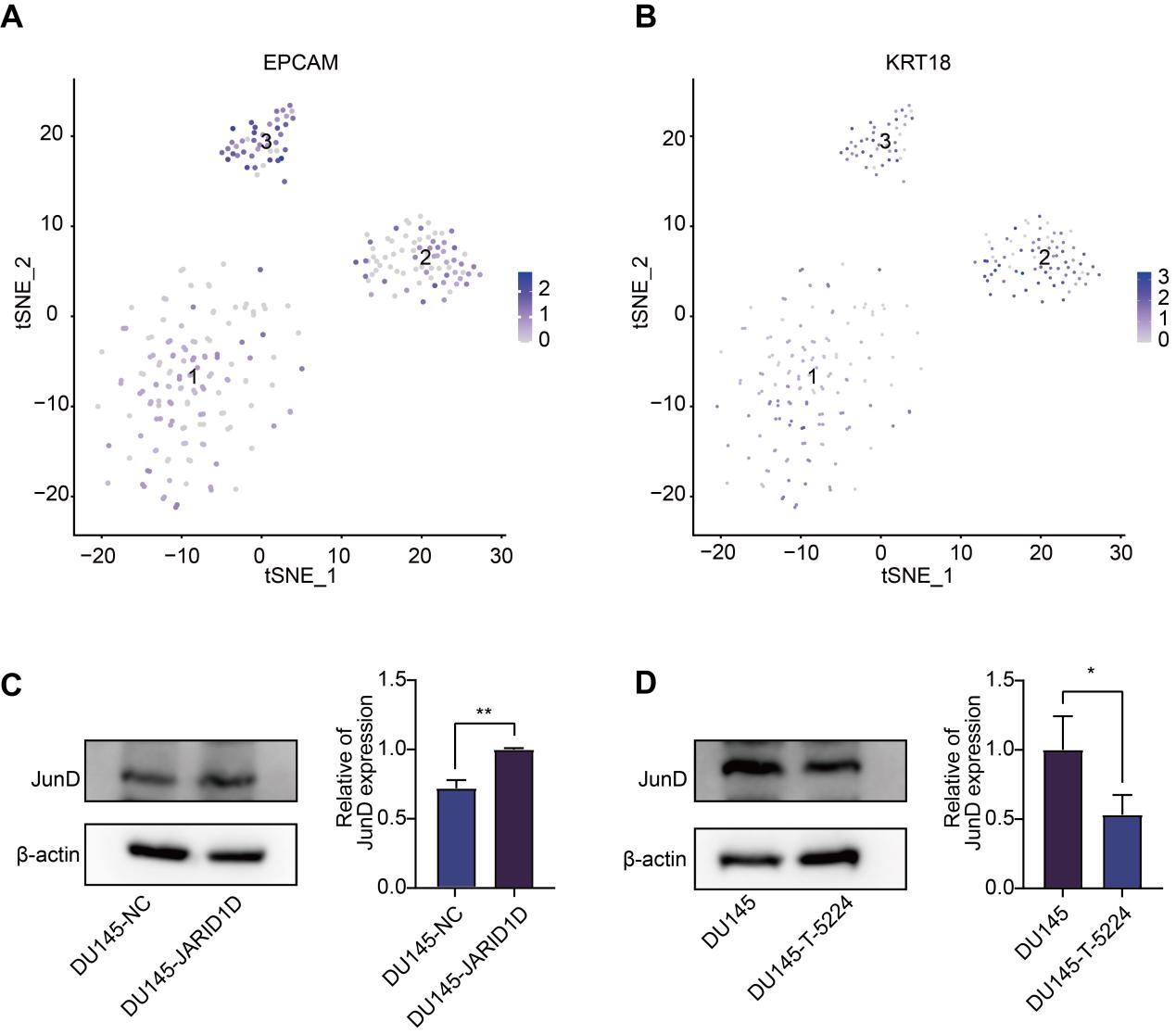


**Supplementary Figure. 5** (A-B) Single-cell omics data from human tumor bone metastases show the detection of EPCAM and KRT18 mRNA in tumor epithelial cell populations; (C) Western Blot and quantitative results indicate that after knocking down JARID1D in DU145 cells, the expression of JunD is increased; (D) Western Blot and quantitative results show that after treating DU145 cells with a JunD inhibitor (T-5224), the expression of JunD decreases.


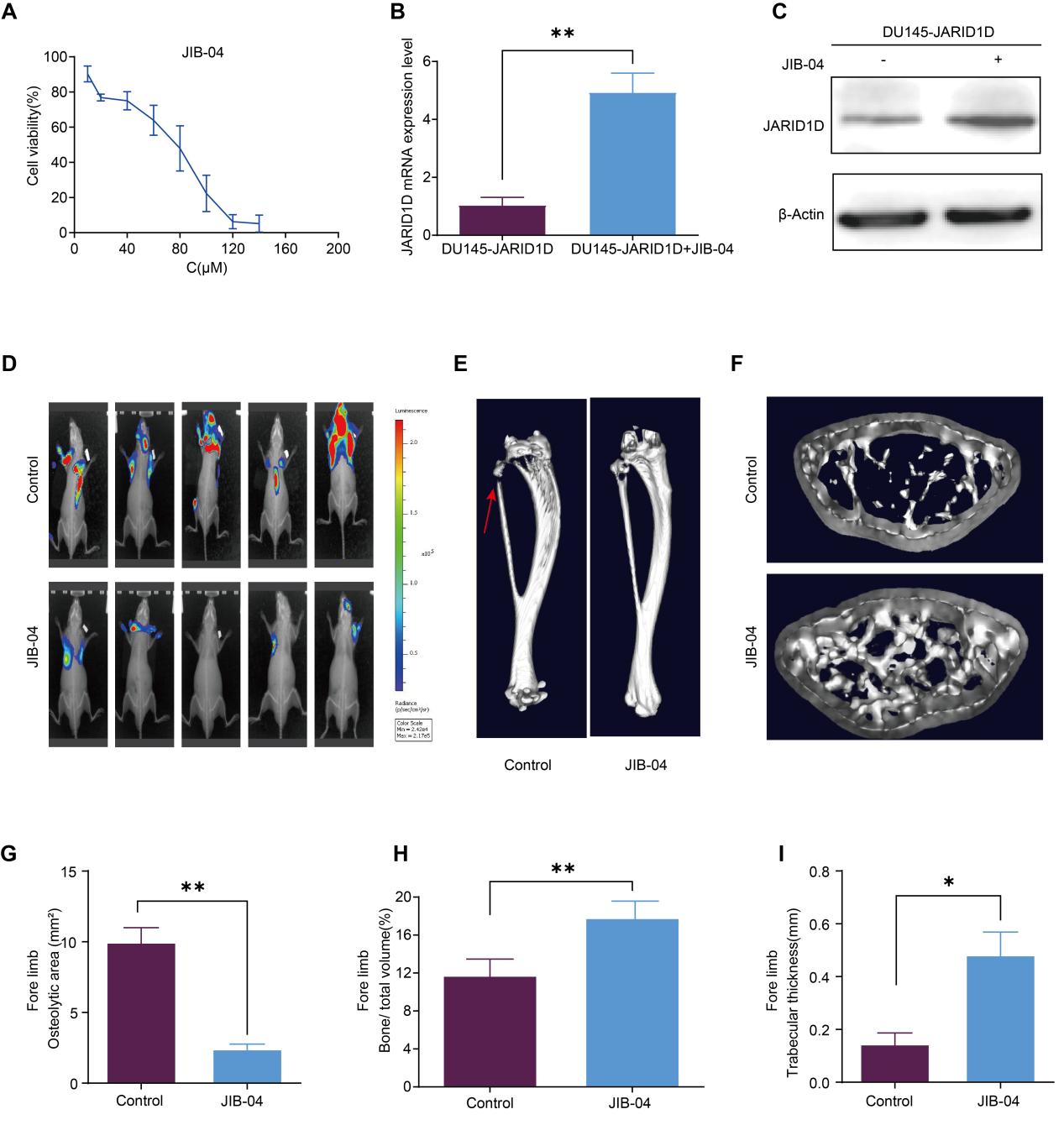


**Supplementary Figure. 6** (A) Effects of JIB-04 on the growth of DU145 cells were analyzed; (B-C) Quantitative RT-PCR (B) and western blotting (C) were used to analyze expression of JARID1D after treatment with JIB-04 in DU145-JARID1D cells; (D) X-ray images of nude mice after JIB-04 treatment; (E) Micro-CT image of tibia of hindlimb of representative nude mouse after JIB-04 treatment; (F) Cross-section of tibia of hindlimb of representative nude mouse after JIB-04 treatment; (G-I) Quantitative map of osteolytic area (G), relative bone volume (H), and trabecular thickness (I) of tibia of hindlimb in (E, F) above (n = 3).


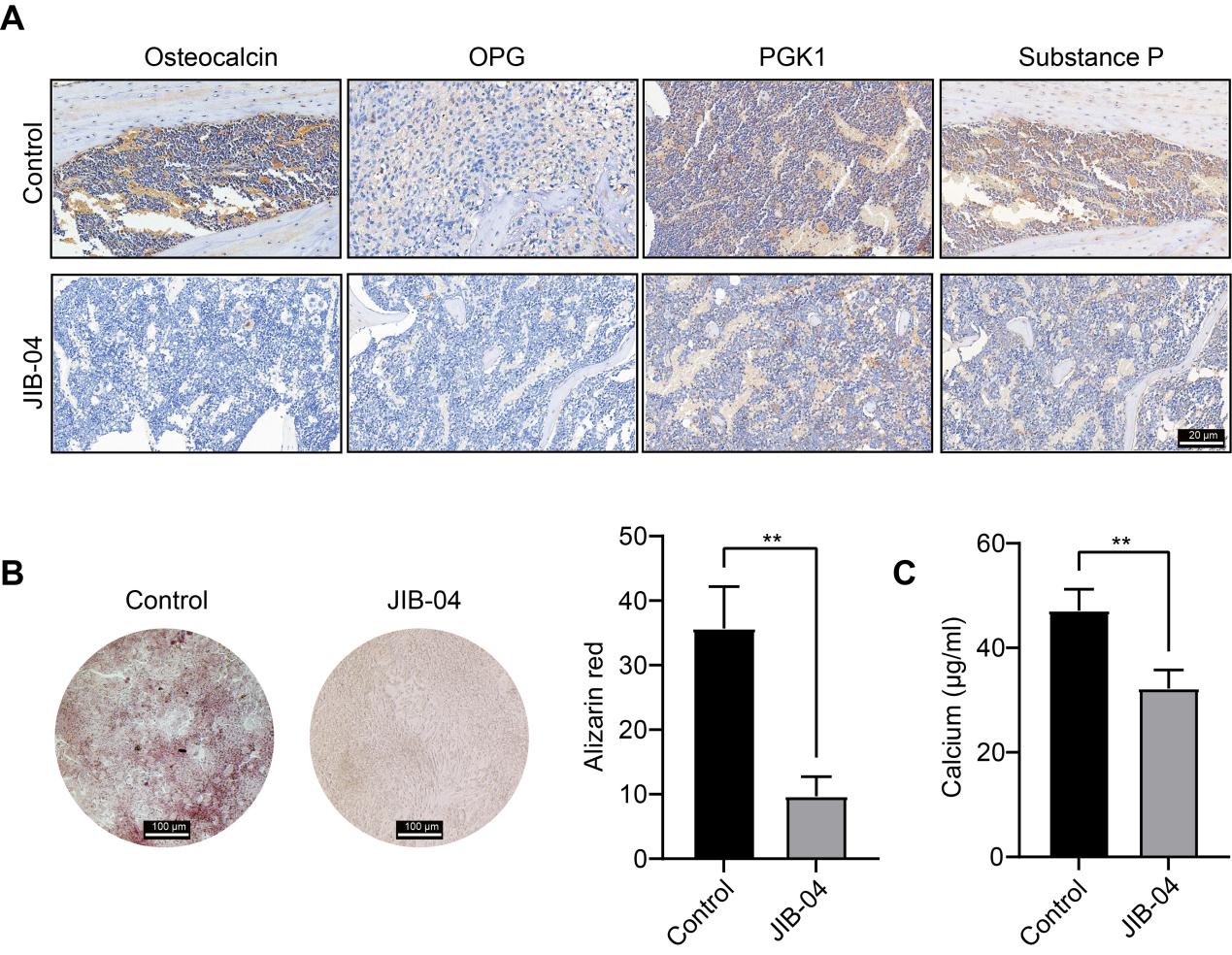


**Supplementary Figure. 7** (A) Representative IHC images showing the expression of osteocalcin and three reported osteoblastic prostate cancer bone metastasis markers, OPG, PGK1, and Substance P in mice from the control and JIB-04 treatment groups. Scale bar: 20 μm; (B) Mouse embryonic osteoblasts MC3T3-E1 subclone 14 cells were cultured with JIB-04 for 16 days, followed by an Alizarin Red S staining assay. The figures show representative wells from each group as well as the relative absorbance of Alizarin Red S; (C) Measurement of calcium content in media from each group (n=3) in (B).

**Supplementary table**

**Supplementary table. 1** **Clinical data of PCa patients**

| Number | Age (year) | PSA value (ng/ml) | Gleason score(points) | TNM | Metastasis site | Treatment |
| --- | --- | --- | --- | --- | --- | --- |
| E97792 | 57 | 22.48 | 4+4=8 | T2bN0M0 | No metastasis | PCa radical surgery |
| D17225 | 64 | 41.58 | 5+4=9 | T3aN1M0 | Tumor invasion of nerves, bladder neck opening, and urethral incision edge PCa radical surgery for cancer tissue | PCa radical surgery |
| E49663 | 67 | 64.65 | 5+5=10 | T4N2M1 | Right acetabulum, ischial bone metastasis, multiple pelvic enlargement, slightly enlarged lymph nodes, partial fusion of bicalutamide and goserelin | Bicalutamide+Goserellin |

**Supplementary table. 2 Primer sequences for Real-time PCR**

| Gene name | Sequence |
| --- | --- |
| JARID1D | F: 5’-AGCCAACCATGTGCAATGTA-3’ |
|  | R: 5’-GGCTCTGGATCAGGCTGTAG-3’ |
| AR | F:5’- AGCTCAAGGATGGAAGTGCAGTTA-3’ |
|  | R: 5’-AATGCTTCACTGGGTGTGGAAATAG-3’ |
| MMP2 | F: 5’-CTGGGAGCATGGCGATGGATA-3’ |
|  | R: 5’-GGAAGCGGAATGGAAACTTG-3’ |
| MMP7 | F: 5’-GCTGACATCATGATTGGCTTTG-3’ |
|  | R: 5’-AGACTGCTACCATCCGTCCA-3’ |
| MMP9 | F: 5’-TTGACAGCGACAAGAAGTGG-3’ |
|  | R: 5’-GCCATTCACGTCGTCCTTAT-3’ |
| Slug | F: 5’-GGGGAGAAGCCTTTTTCTTG-3’ |
|  | R: 5’-TCCTCATGTTTGTGCAGGAG-3’ |
| Snail | F: 5’-CCTCCCTGTCAGATGAGGAC-3’ |
|  | R: 5’-CCAGGCTGAGGTATTCCTTG-3’ |
| N-Cadherin | F: 5’-CAATGCCGCCATCGCTTAC-3’ |
|  | R: 5’-ATGACTCCTGTGTTCCTGTTAATG-3’ |
| Vimentin | F: 5’-GAGAACTTTGCCGTTGAAGC-3’ |
|  | R: 5’-TCCAGCAGCTTCCTGTAGGT-3’ |
| RANKL | F:5’-CAGTGGGAGATGTTAGACTCATG-3’ |
|  | R:5’-GAAGGGGCACATGACCAGGGACCAAC-3’ |
| TRAP | F:5’-CACTCCCACCCTGAGATTTGTG-3’ |
|  | R:5’-ACGGTTCTGGCGATCTCTTTGC-3’ |
| C-Fos | F:5’-TACTACCATTCCCCAGCCGA-3’ |
|  | R:5’-GCTGTCACCGTGGGGATAAA-3’ |
| Cathepsink | F:5’-TGGTTCACTGGAACACCAAA-3’ |
|  | R:5’-AGCAAGGGTCGAAGTTAGCA-3’ |
| NFATc1 | F:5’- TCATCGGCGGGAAGAAGATG-3’ |
|  | R:5’- GTCCCGGTCAGTCTTTGCTT-3’ |
| AR | F:5’- AGCTCAAGGATGGAAGTGCAGTTA-3’ |
|  | R: 5’-AATGCTTCACTGGGTGTGGAAATAG-3’ |
| IL-6 | F:5’- CAAGAGACTTCCATCCAGTTGCCT-3’ |
|  | R: 5’-TTTCTCATTTCCACGATTTCCCAG-3’ |
| ENO | F:5’GCCAAAGGTCTTTTCCGG-3’ |
|  | R: 5’-CCTTCAGGACACCTTTGC-3’ |
| CGA | F:5’ATACCAGGTGATGAAATGC-3’ |
|  | R: 5’-AGGATCCGTTCATCTCCTC-3’ |
| COL1A1 | F:5’- GTGCGATGACGTGATCTGTGA-3’ |
|  | F:5’- CGGTGGTTTCTTGGTCGGT-3’ |
| SOX9 | F:5’- CCCGCTCACAGTACGACTAC-3’ |
|  | R:5’- CTGAGCGGGGTTCATGTAGG-3’ |
| HSD17B4 | F:5’- TTGGGCCGAGCCTATGC-3’ |
|  | R:5’- CCCCTCCCAAATCATTCACA-3’ |
| SEMA4D | F:5’- AATGTTTGACGACACTGATGGT-3’ |
|  | R:5’- TCTTTGCTGGTGCTAGAGATG-3’ |
| RUNX1 | F:5’- TGAGCTGAGAAATGCTACCGC-3’ |
|  | R:5’- ACTTCGACCGACAAACCTGAG-3’ |
| COL1A2 | F:5’- GGTGAAGTGGGTCTTCCAGG-3’ |
|  | R:5’- TAAGGCCGTTTGCTCCAGG-3’ |
| JunD | F:5’- ATCGACATGGACACGCAGGAGC-3’ |
|  | R:5’- CTCCGTGTTCTGACTCTTGAGG-3’ |
| MGP | F:5’- AGAACGCTCTAAGCCTGTCCA-3’ |
|  | R:5’- GGCAGCATTGTATCCATAAACC-3’ |
| JUNB | F:5’- TCTTTGCTGGTGCTAGAGATG-3’ |
|  | R:5’- CGAGTTCTGAGCTTTCAAGGT-3’ |
| MAOA | F: 5’-CTGATCGACTTGCTAAGCTAC-3’ |
|  | R: 5’-ATGCACTGGATGTAAAGCTTC-3’ |
| OPG | F: 5’-AACCCCAGAGCGAAATAC-3’ |
|  | R: 5’-AAGAATGCCTCCTCACAC-3’ |
| PGK1 | F: 5’-CTGTGGGGGTATTTGAATGG-3’ |
|  | R: 5’-CTGTGGGGGTATTTGAATGG-3’ |
| Substance P | F: 5’-GTACGACAGCGACCAGATCA-3’ |
|  | R: 5’-AGCCTTTAACAGGGCCACTT-3’ |
| EMID1 | F: 5’-TAAGGGAGACCCTGGTGAGA-3’ |
|  | R: 5’-GACCCCAGCTCTGGTTCATA-3’ |
| β-actin | F: 5’-CATGTACGTTGCTATCCAGGC-3’ |
|  | R: 5’-CTCCTTAATGTCACGCACGAT-3’ |
