# Supplementary material for: JARID1D-dependent androgen receptor and JunD signaling activation of osteoclast differentiation inhibits prostate cancer bone metastasis through demethylating H3K4
Source: Theranostics. 2025 Jan 1;15(4):1320–37. doi: 10.7150/thno.104135 (PMC11729558; doi:10.7150/thno.104135)
Supplement: Supplementary file 1 — Supplementary figures and tables. [file thnov15p1320s1.zip › Supplementary-Meterial/Supplementary-Material.pdf]

Supplementary Material

JARID1D-dependent androgen receptor and JunD signaling activation of osteoclast differentiation inhibits prostate cancer bone metastasis through demethylating H3K4

Authors' names: Yaohua Hu, Zhite Zhao, Qinghua Xie, Hui Li, Chenyang Zhang, Xinglin He, Yifan Ma, Caiqin Zhang, Qinlong Li

*Correspondence:

Changhong Shi

Division of Cancer Biology, Laboratory Animal Center, The Fourth Military Medical University, Xi'an, Shaanxi 710032, China

Tel: 86-29-84774787

Fax: 86-29-83242045

Email: changhong@fmmu.edu.cn

Qinlong Li

Department of Pathology, School of Basic Medicine and Xijing Hospital, State Key Laboratory of Cancer Biology, The Fourth Military Medical University, Xi'an, China.

qinlongli@163.com

Supplementary Figure

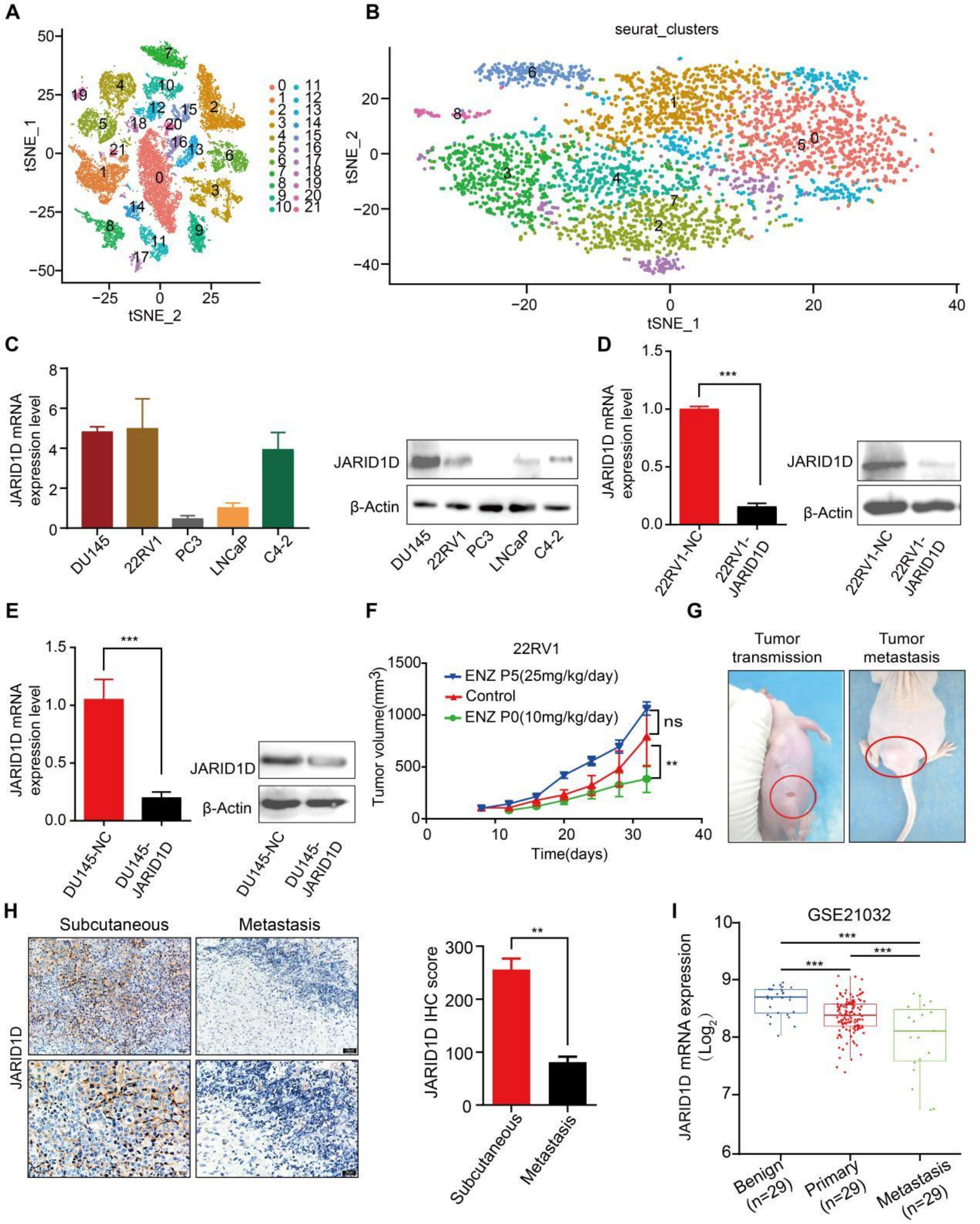

J

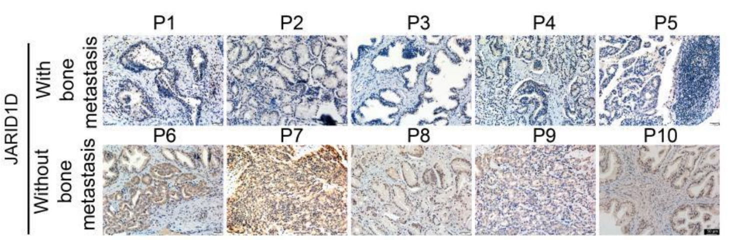

IHC score \ Group	With bone metastasis	Without bone metastasis
1	N=16	N=2
2	N=3	N=3
3	N=0	N=12
4	N=1	N=3

Supplementary Figure. 1 (A) A t-SNE plot showing 28 cell clusters by unsupervised graph clustering; (B) A t-SNE plot of JARID1D showing normalized expression level in the sub-clusters; (C) Expression levels of JARID1D in prostate cancer cell lines; (D-E) Expression of JARID1D after 22RV1 (D) and DU145 (E) cells was transfected into lentivirus was analyzed using quantitative RT-PCR and western blotting; (F) The tumor growth curves of mice in the Control group, ENZ P0 group and ENZ P5 group; (G) Tumor Metastasis Site Photographs; (H) The expression of JARID1D in subcutaneous and metastatic tumors in mice; (I) JARID1D mRNA levels in PCa tissues were assessed by analyzing the GS21032; (J) IHC analysis of primary prostate cancer samples from 40 patients with and without bone metastasis. Each sample was scored based on the percentage of JARID1D positivity (1 points: 0-25%, 2 points: 26-50%, 3 points: 51-75%, 4 points: 76-100%). Scale bar: 20 μm.

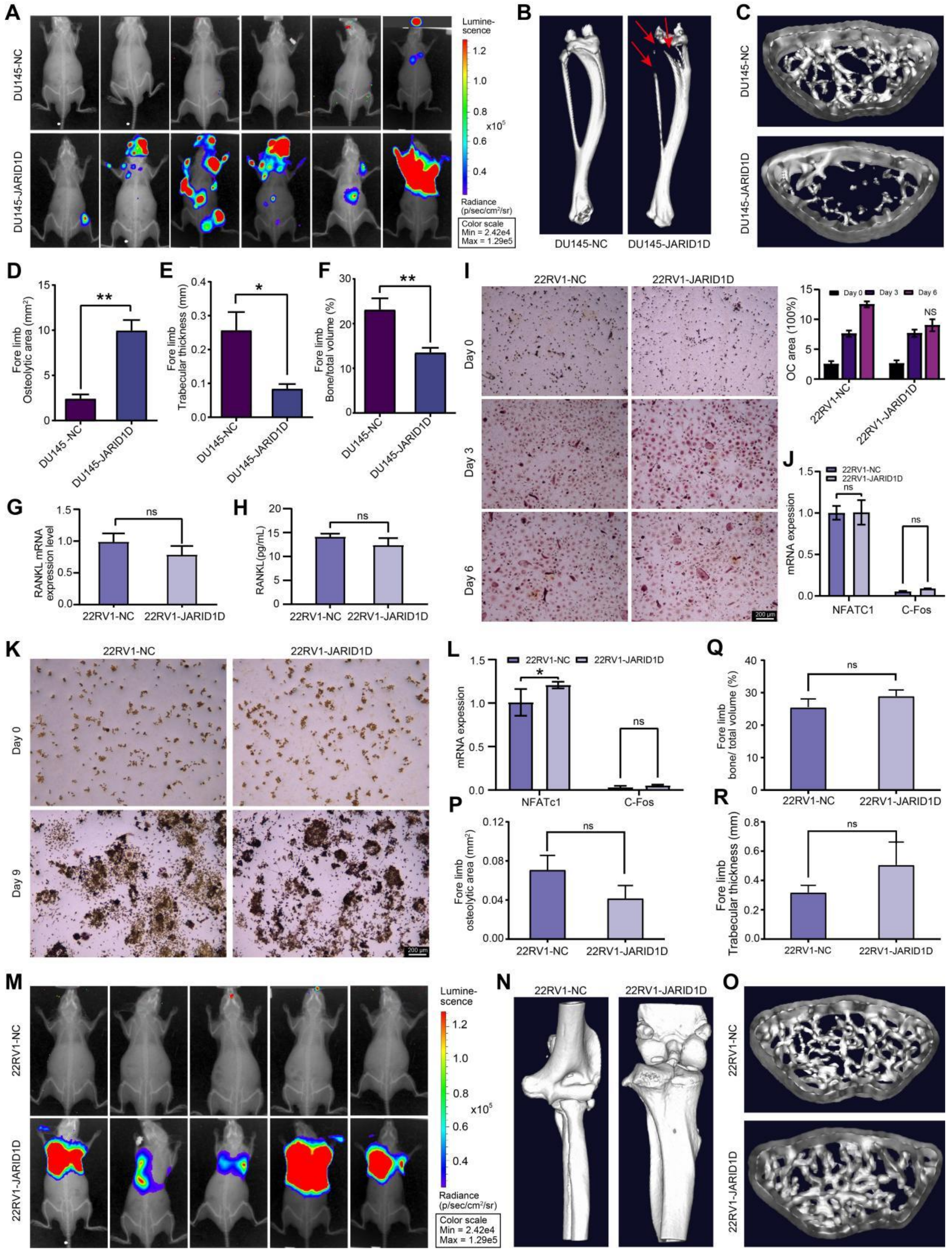

Supplementary Figure. 2 (A) X-ray image of DU145-NC and DU145 Sh-JARID1D cells intracardially injected into nude mice; (B) Micro-CT image of tibia of hindlimb of representative nude mouse in DU145-NC and DU145 sh-JARID1D groups; (C) Cross section of tibia of hindlimb of representative

nude mouse in DU145-NC and DU145 Sh-JARID1D groups; (D-F) Quantitative map of osteolytic area (D) relative bone volume (E) and trabecular thickness (F) of tibia of hindlimb in (A) above (n = 3); (G) RT-PCR detection of RANKL expression in 22RV1 cells with JARID1D knockdown; (H) ELISA detection of RANKL content in the culture supernatant of 22RV1cells after JARID1D knockdown; (I) TRAP staining to assess the differentiation capacity of osteoclasts in different treatment groups and quantification results; (J) RT-PCR analysis of osteoclast differentiation-related gene expression across various treatment groups; (K) Representative TRAP staining images and quantification results of osteoclast differentiation induced by RAW264.7 cell in different treatment groups; (L) RT-PCR analysis of osteoclast differentiation-related gene expression across various treatment groups; (M) X-ray image of 22RV1-NC and 22RV1 Sh-JARID1D cells intracardially injected into nude mice; (N) Micro-CT image of tibia of hindlimb of representative nude mouse in 22RV1-NC and 22RV1Sh-JARID1D groups. (O) Cross section of tibia of hindlimb of representative nude mouse in DU145-NC and DU145 sh-JARID1D groups. (P-R) Quantitative map of osteolytic area (P) relative bone volume (Q) and trabecular thickness (R) of tibia of hindlimb in (M, N) above (n = 3).

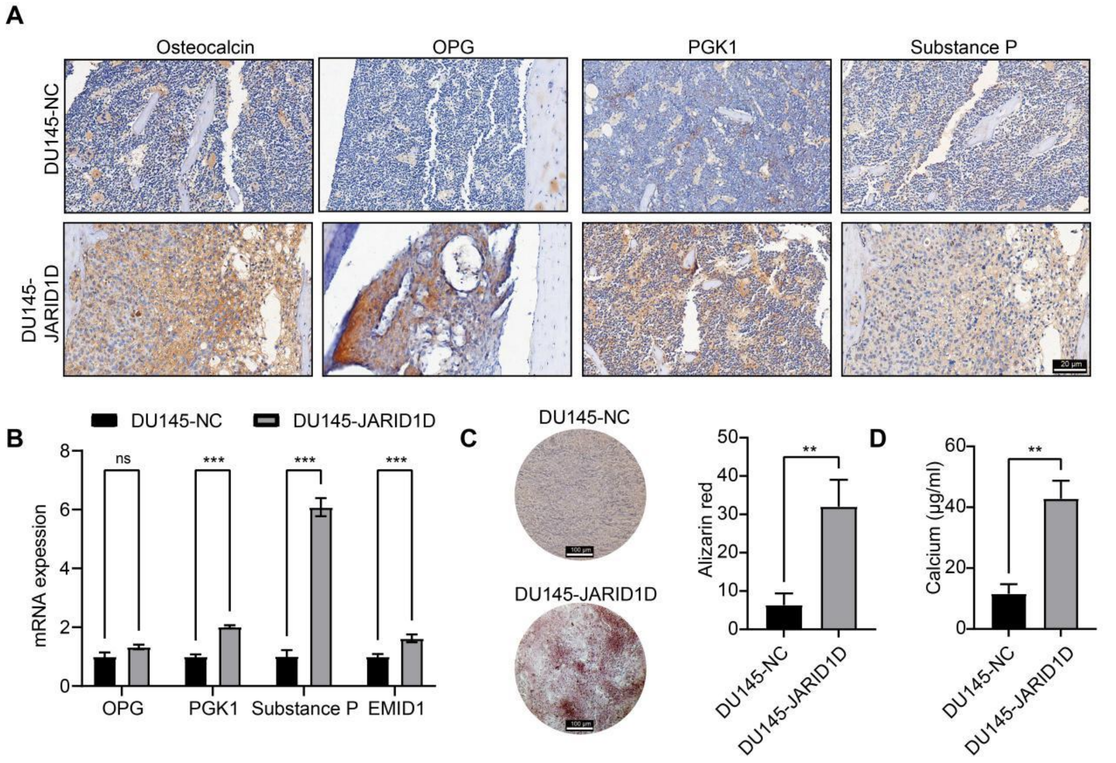

Supplementary Figure. 3 (A) Representative IHC images of osteocalcin and three reported osteoblastic prostate cancer bone metastasis markers, OPG, PGK1, and Substance P, in tumor-associated osteoblasts from bone metastases in mice inoculated with DU145-NC and DU145-ShJARID1D cells. Scale bar: 20 μm; (B) mRNA expression of 4 reported markers of osteoblastic PCa bone metastases, OPG, PGK1, Substance P and EMID1, in tumor cells by RT-qPCR; (C) mouse embryonic osteoblasts MC3T3-E1 subclone 14 cells were cultured for 16 days in DU145-NC or DU145-ShJARID1D conditioned media, followed by an Alizarin Red S staining assay. The figures show representative wells from each group as well as the relative absorbance of Alizarin Red S; (D) Measurement of calcium content in media from each group (n=3) in (C) .

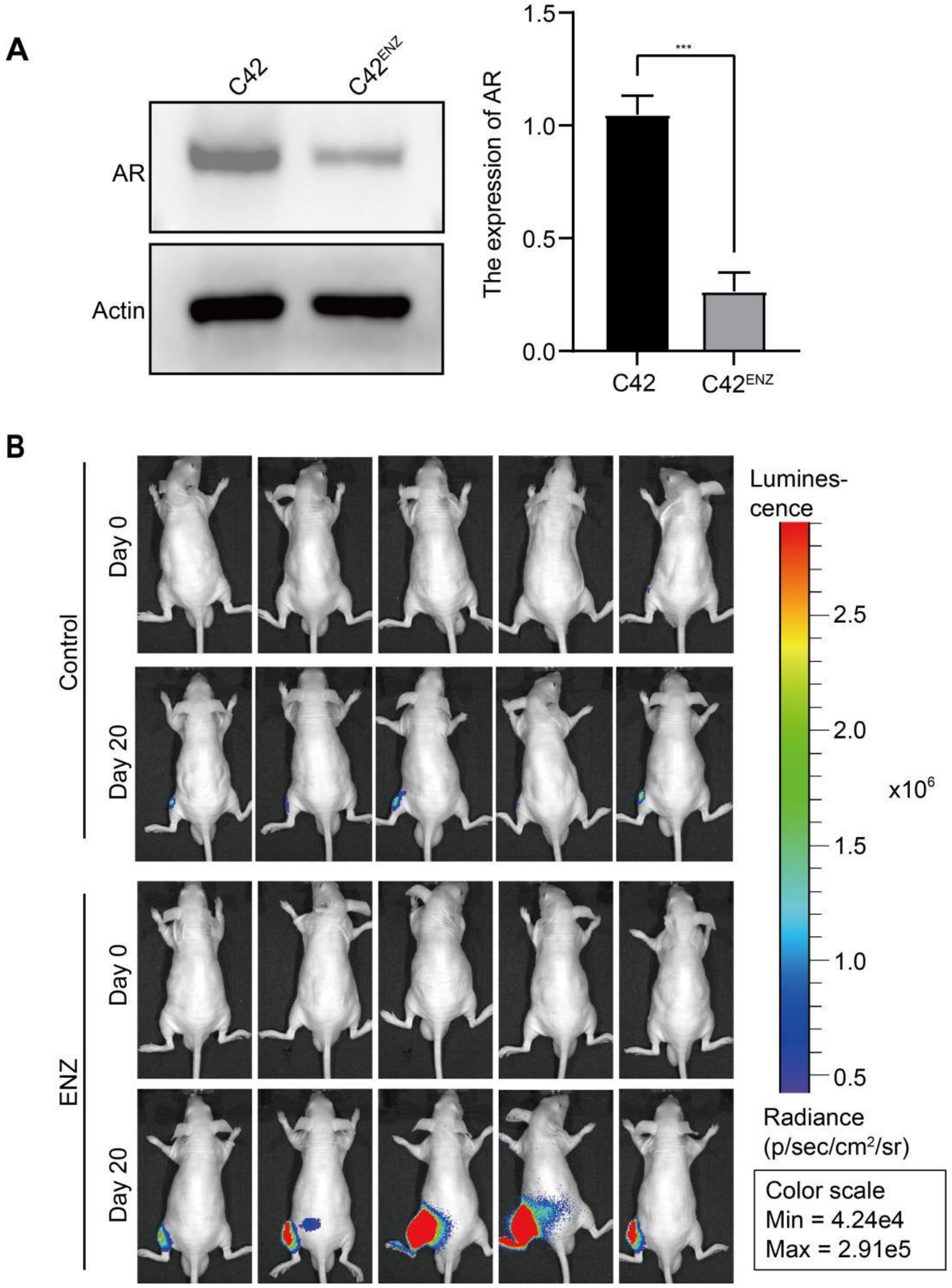

Supplementary Figure. 4 (A) The protein expression levels of AR in C42 and C42^{ENZ} (induced for one year with 20 μm ENZ); (B) Representative images of in vivo imaging in small animals.from different treatment groups on Day 0 and Day 20.

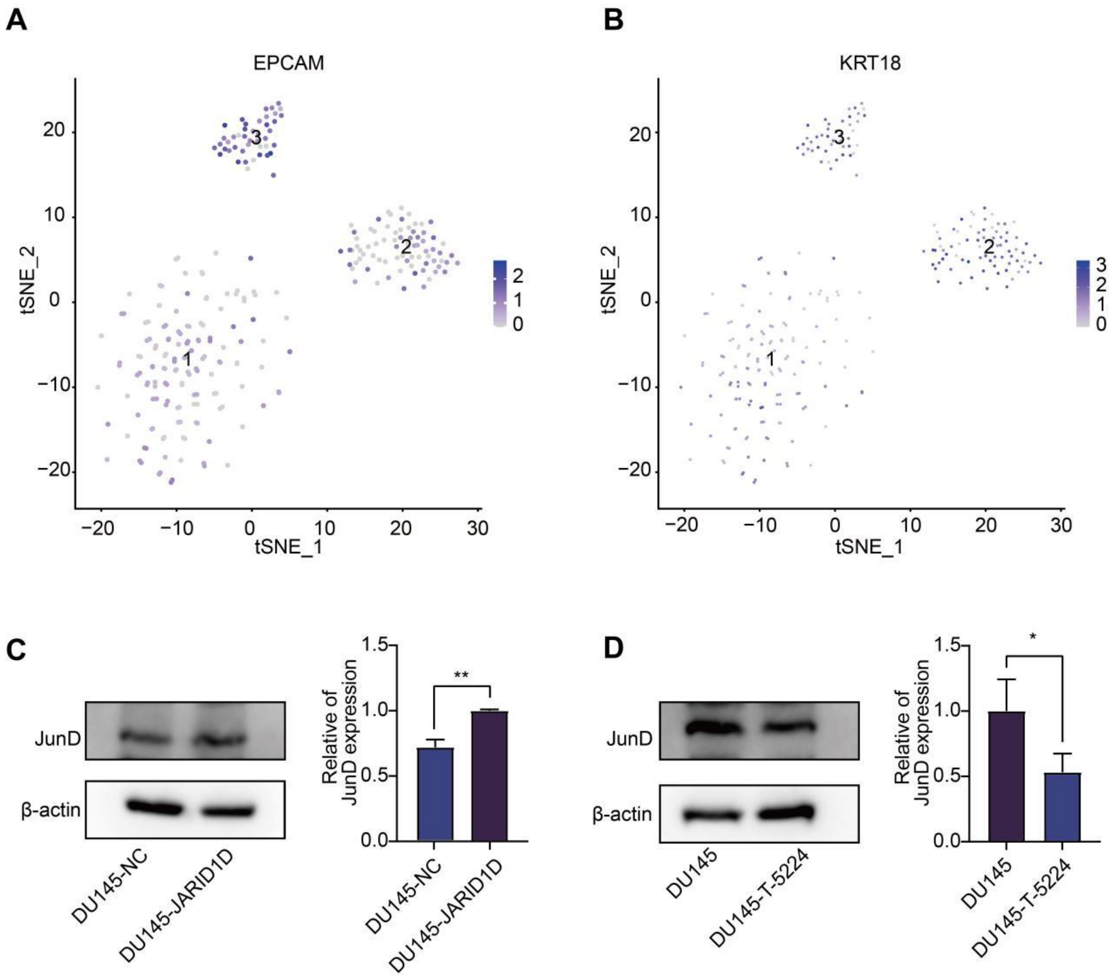

Supplementary Figure. 5 (A-B) Single-cell omics data from human tumor bone metastases show the detection of EPCAM and KRT18 mRNA in tumor epithelial cell populations; (C) Western Blot and quantitative results indicate that after knocking down JARID1D in DU145 cells, the expression of JunD is increased; (D) Western Blot and quantitative results show that after treating DU145 cells with a JunD inhibitor (T-5224), the expression of JunD decreases.

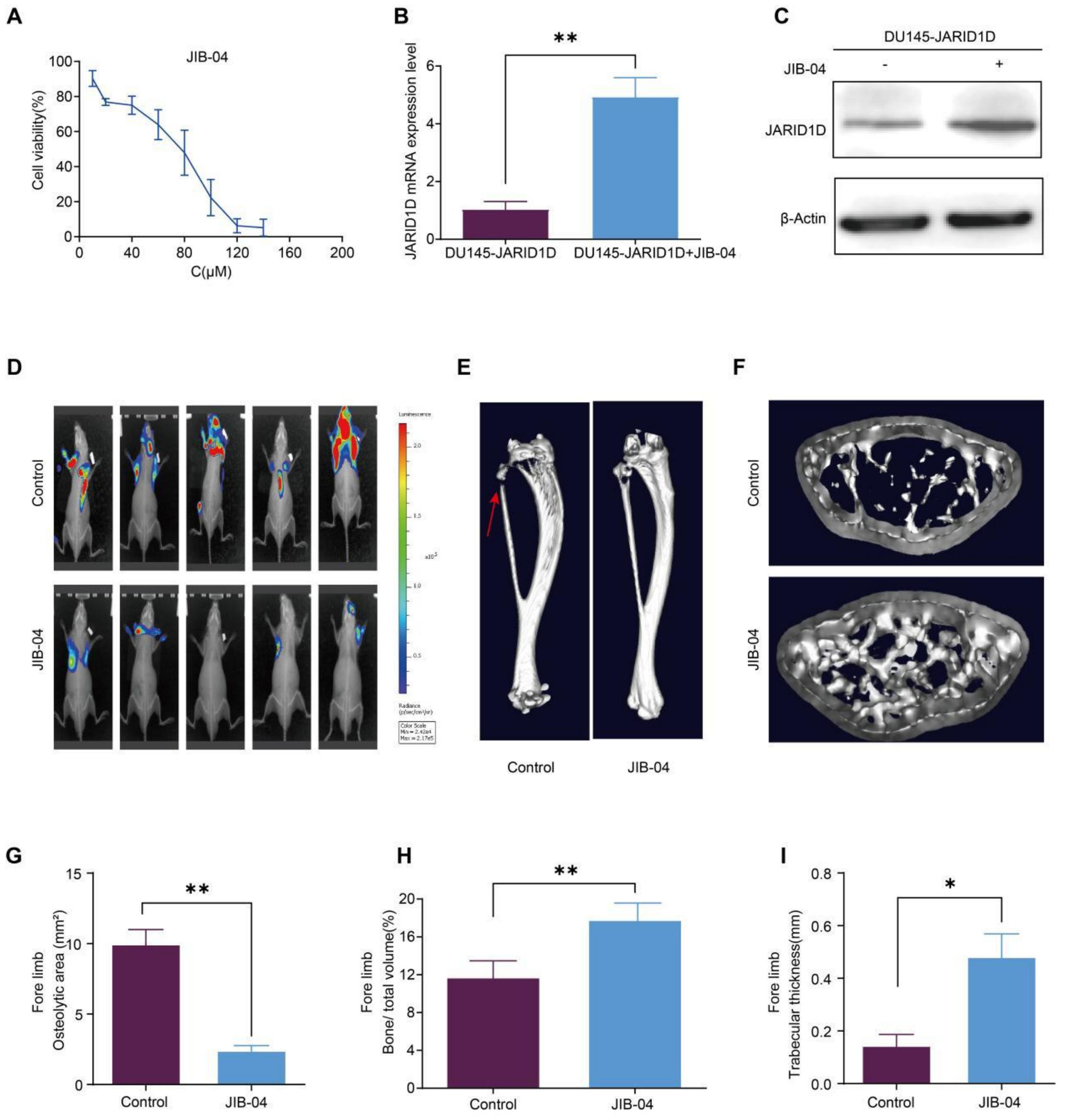

Supplementary Figure. 6 (A) Effects of JIB-04 on the growth of DU145 cells were analyzed; (B-C) Quantitative RT-PCR (B) and western blotting (C) were used to analyze expression of JARID1D after treatment with JIB-04 in DU145-JARID1D cells; (D) X-ray images of nude mice after JIB-04 treatment; (E) Micro-CT image of tibia of hindlimb of representative nude mouse after JIB-04 treatment; (F) Cross-section of tibia of hindlimb of representative nude

mouse after JIB-04 treatment; (G-I) Quantitative map of osteolytic area (G), relative bone volume (H), and trabecular thickness (I) of tibia of hindlimb in (E, F) above (n = 3).

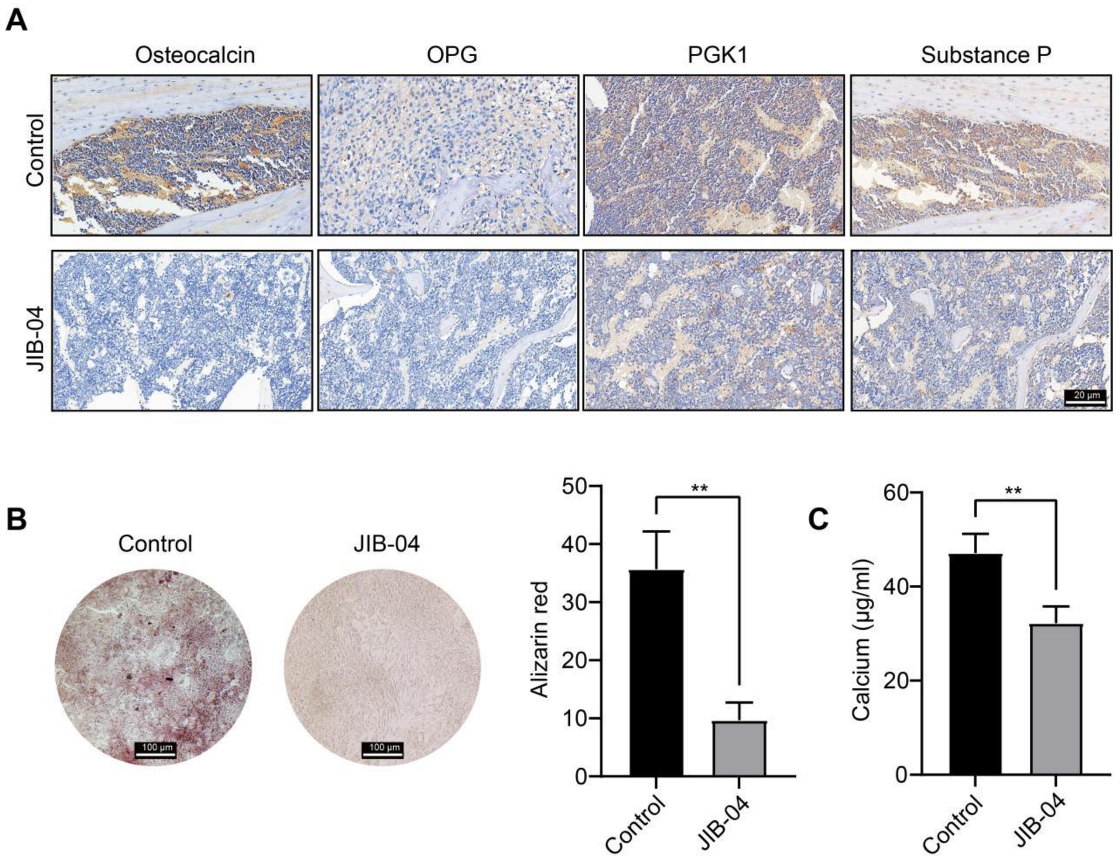

Supplementary Figure. 7 (A) Representative IHC images showing the expression of osteocalcin and three reported osteoblastic prostate cancer bone metastasis markers, OPG, PGK1, and Substance P in mice from the control and JIB-04 treatment groups. Scale bar: 20 μm; (B) Mouse embryonic osteoblasts MC3T3-E1 subclone 14 cells were cultured with JIB-04 for 16 days, followed by an Alizarin Red S staining assay. The figures show representative wells from each group as well as the relative absorbance of Alizarin Red S; (C) Measurement of calcium content in media from each group (n=3) in (B).

Supplementary table

Supplementary table. 1 Clinical data of PCa patients

Number	Age (year)	PSA value (ng/ml)	Gleason score(points)	TNM	Metastasis site	Treatment
E97792	57	22.48	4+4=8	T2bN0M0	No metastasis	PCa radical surgery
D17225	64	41.58	5+4=9	T3aN1M0	Tumor invasion of nerves, bladder neck opening, and urethral incision edge PCa radical surgery for cancer tissue	PCa radical surgery
E49663	67	64.65	5+5=10	T4N2M1	Right acetabulum, ischial bone metastasis, multiple pelvic enlargement, slightly enlarged lymph nodes, partial fusion of bicalutamide and goserelin	Bicalutamide+Goserellin

Supplementary table. 2 Primer sequences for Real-time PCR

Gene name	Sequence
JARID1D	F: 5'-AGCCAACCATGTGCAATGTA-3'<br>R: 5'-GGCTCTGGATCAGGCTGTAG-3'
AR	F:5'- AGCTCAAGGATGGAAGTGCAGTTA-3'<br>R: 5'-AATGCTTCACTGGGTGTGGAAATAG-3'
MMP2	F: 5'-CTGGGAGCATGGCGATGGATA-3'<br>R: 5'-GGAAGCGGAATGGAAACTTG-3'
MMP7	F: 5'-GCTGACATCATGATTGGCTTTG-3'<br>R: 5'-AGACTGCTACCATCCGTCCA-3'
MMP9	F: 5'-TTGACAGCGACAAGAAGTGG-3'<br>R: 5'-GCCATTCACGTCGTCCTTAT-3'
Slug	F: 5'-GGGGAGAAGCCTTTTTCTTG-3'<br>R: 5'-TCCTCATGTTTGTGCAGGAG-3'
Snail	F: 5'-CCTCCCTGTCAGATGAGGAC-3'<br>R: 5'-CCAGGCTGAGGTATTCCTTG-3'
N-Cadherin	F: 5'-CAATGCCGCCATCGCTTAC-3'<br>R: 5'-ATGACTCCTGTGTTCCTGTTAATG-3'
Vimentin	F: 5'-GAGAACTTTGCCGTTGAAGC-3'<br>R: 5'-TCCAGCAGCTTCCTGTAGGT-3'
RANKL	F:5'-CAGTGGGAGATGTTAGACTCATG-3'

	R:5’-GAAGGGGCACATGACCAGGGACCAAC-3’
TRAP	F:5’-CACTCCCACCCTGAGATTTGTG-3’<br>R:5’-ACGGTTCTGGCGATCTCTTTGC-3’
C-Fos	F:5’-TACTACCATTCCCCAGCCGA-3’<br>R:5’-GCTGTCACCGTGGGGATAAA-3’
Cathepsink	F:5’-TGGTTCACTGGAACACCAAA-3’<br>R:5’-AGCAAGGGTCGAAGTTAGCA-3’
NFATc1	F:5’- TCATCGGCGGGAAGAAGATG-3’<br>R:5’- GTCCCGGTCAGTCTTTGCTT-3’
AR	F:5’- AGCTCAAGGATGGAAGTGCAGTTA-3’<br>R: 5’-AATGCTTCACTGGGTGTGGAAATAG-3’
IL-6	F:5’- CAAGAGACTTCCATCCAGTTGCCT-3’<br>R: 5’-TTTCTCATTTCCACGATTTCCCAG-3’
ENO	F:5’GCCAAAGGTCTTTTCCGG-3’<br>R: 5’-CCTTCAGGACACCTTTGC-3’
CGA	F:5’ATACCAGGTGATGAAATGC-3’<br>R: 5’-AGGATCCGTTCATCTCCTC-3’
COL1A1	F:5’- GTGCGATGACGTGATCTGTGA-3’<br>F:5’- CGGTGGTTTCTTGGTCGGT-3’
SOX9	F:5’- CCCGCTCACAGTACGACTAC-3’<br>R:5’- CTGAGCGGGGTTCATGTAGG-3’
HSD17B4	F:5’- TTGGGCCGAGCCTATGC-3’

	R:5’- CCCCTCCCAAATCATTCACA-3’
SEMA4D	F:5’- AATGTTTGACGACACTGATGGT-3’<br>R:5’- TCTTTGCTGGTGCTAGAGATG-3’
RUNX1	F:5’- TGAGCTGAGAAATGCTACCGC-3’<br>R:5’- ACTTCGACCGACAAACCTGAG-3’
COL1A2	F:5’- GGTGAAGTGGGTCTTCCAGG-3’<br>R:5’- TAAGGCCGTTTGCTCCAGG-3’
JunD	F:5’- ATCGACATGGACACGCAGGAGC-3’<br>R:5’- CTCCGTGTTCTGACTCTTGAGG-3’
MGP	F:5’- AGAACGCTCTAAGCCTGTCCA-3’<br>R:5’- GGCAGCATTGTATCCATAAACC-3’
JUNB	F:5’- TCTTTGCTGGTGCTAGAGATG-3’<br>R:5’- CGAGTTCTGAGCTTTCAAGGT-3’
MAOA	F: 5’-CTGATCGACTTGCTAAGCTAC-3’<br>R: 5’-ATGCACTGGATGTAAAGCTTC-3’
OPG	F: 5’-AACCCCAGAGCGAAATAC-3’<br>R: 5’-AAGAATGCCTCCTCACAC-3’
PGK1	F: 5’-CTGTGGGGGTATTTGAATGG-3’<br>R: 5’-CTGTGGGGGTATTTGAATGG-3’
Substance P	F: 5’-GTACGACAGCGACCAGATCA-3’<br>R: 5’-AGCCTTTAACAGGGCCACTT-3’
EMID1	F: 5’-TAAGGGAGACCCTGGTGAGA-3’

	R: 5’-GACCCCAGCTCTGGTTCATA-3’
β-actin	F: 5’-CATGTACGTTGCTATCCAGGC-3’
	R: 5’-CTCCTTAATGTCACGCACGAT-3’
